# A confinable female-lethal population suppression system in the malaria vector, *Anopheles gambiae*

**DOI:** 10.1126/sciadv.ade8903

**Published:** 2023-07-05

**Authors:** Andrea L. Smidler, James J. Pai, Reema A. Apte, Héctor M. Sánchez C., Rodrigo M. Corder, Eileen Jeffrey Gutiérrez, Neha Thakre, Igor Antoshechkin, John M. Marshall, Omar S. Akbari

**Affiliations:** ^1^ School of Biological Sciences, Department of Cell and Developmental Biology, University of California San Diego, La Jolla, CA 92093, USA.; ^2^ Divisions of Epidemiology and Biostatistics, School of Public Health, University of California, Berkeley, CA 94720, USA.; ^3^ Oxitec Ltd., Abingdon, OX14 4RQ, UK.; ^4^ Division of Biology and Biological Engineering (BBE), California Institute of Technology, Pasadena, CA 91125, USA.; ^5^ Innovative Genomics Institute, University of California, Berkeley, CA 94720, USA.

## Abstract

Malaria is among the world’s deadliest diseases, predominantly affecting Sub-Saharan Africa and killing over half a million people annually. Controlling the principal vector, the mosquito *Anopheles gambiae*, as well as other anophelines, is among the most effective methods to control disease spread. Here, we develop a genetic population suppression system termed Ifegenia (inherited female elimination by genetically encoded nucleases to interrupt alleles) in this deadly vector. In this bicomponent CRISPR-based approach, we disrupt a female-essential gene, *femaleless* (*fle*), demonstrating complete genetic sexing via heritable daughter gynecide. Moreover, we demonstrate that Ifegenia males remain reproductively viable and can load both *fle* mutations and CRISPR machinery to induce *fle* mutations in subsequent generations, resulting in sustained population suppression. Through modeling, we demonstrate that iterative releases of nonbiting Ifegenia males can act as an effective, confinable, controllable, and safe population suppression and elimination system.

## Teaser

Ifegenia is a GM vector control system for female killing and genetic sexing of the African malaria mosquito, *Anopheles gambiae*.

## INTRODUCTION

Anopheline mosquitoes are responsible for malaria transmission, with the *Anopheles gambiae* complex being the most dangerous, contributing to a quarter billion annual cases (*1*). Controlling anophelines is one of the most effective strategies to prevent disease transmission. However, existing suppression tools such as insecticides and bed nets are becoming increasingly ineffective (*2*). Furthermore, plastic mosquito behaviors may be biasing toward exophagy (*3*), both contributing to the plateau in disease reduction and increasing the costs of control (*1*). Therefore, the development of sustainable, efficient, safe, scalable, and cost-effective vector control technologies is urgently needed.

Current vector control methods are limited to habitat removal and insecticide-based measures; however, control methods relying on direct release of modified mosquitoes are gaining traction. Because only female mosquitoes transmit disease, most vector control campaigns require exclusive releases of nonbiting males. Producing sufficient males en masse necessitates development of mechanical, chemical, or genetic sex-sorting mechanisms (*4*). Unlike other mosquitoes, sex separation by pupal size is not considered possible in *A. gambiae* (*5*). Moreover, lines that permit sex sorting via insecticide resistance are counter-recommended (*6*), and feeding larvae female-killing RNA interference yields incomplete phenotypes (*7*), suggesting that transgenic methods may be more robust. Directly sorting males is possible by genetically encoding fluorescence on sex chromosomes, near the sex-determining loci (*8*), or by using sex-specific alternative splicing or promoters (*9*–*11*). However, these methods often require each released male be directly sorted, which is labor-intensive, requiring sorting facilities near release sites, thus making releases in remote areas exceedingly difficult. In mosquitoes, genetic approaches that can induce female lethality through removal of chemical inhibition (*12*), or by performing genetic crosses, have been developed (*13*). Unfortunately, these technologies have not been adapted to anophelines.

In anophelines, male-biased transgenics have been developed by expressing nucleases targeting the X chromosome during spermatogenesis (*14*). However, these transgenes are constitutively dominant, complicating mass rearing, and show incomplete phenotypes, requiring labor-intensive manual selection before release. Furthermore, field trials using a precursor system exhibit reduced fitness, presumably due to leaky transgene expression in other tissues targeting the male X (*15*), suggesting that an optimal genetic sexing system (GSS) should not target male-essential factors. In another sex-biasing technology, constitutive overexpression of the male-determining factor, *Yob*, causes male bias in *A. gambiae*, but it causes incomplete phenotypes and faces challenges to scale (*16*). Therefore, while these tools are promising, many now available technologies are not scalable as a GSS for *A. gambiae* (*4*). Last, while sex-distorting gene drives have been developed in *A. gambiae* (*17*), these technologies face substantial technical, political, ethical, and regulatory hurdles due to the autonomous nature of their spread, making implementation of this technology challenging (*18*). Furthermore, evidence suggests that these types of suppression drives may be hindered by evolution of resistance alleles (*19*).

Recently, a previously unknown gene in the anopheline sex-determination pathway, *femaleless* (*fle*), was discovered (*20*). Reported to play a role in X-chromosome dosage compensation and splicing of the keystone sex-determination gene, *doublesex* (*dsx*), some experiments suggested a female-lethal characteristic depending on timing of knockdown. To engineer a novel population suppression approach, we developed a binary CRISPR-based vector control technology targeting *fle* (*20*). Because of its profound daughter-killing phenotypes, we have termed it Ifegenia (inherited female elimination by genetically encoded nucleases to interrupt alleles), in honor of Iphigenia of Greek mythology who was sacrificed by her father, King Agamemnon, to win a great battle. Ifegenia operates as a bicomponent approach using distinct Cas9 and guide RNA (gRNA) encoding lines, which result in female lethality upon hybridization, similar to precision-guided sterile insect technique (pgSIT) (*13*, *21*). Offspring incur mosaic somatic and heritable germline *fle* mutations, resulting in early larval daughter gynecide while leaving sibling males reproductively viable. These males can be iteratively released to load populations with mutations in female-essential gene(s) and causative CRISPR transgenes inducing prolonged, nondriving, population suppression, or sterilized for use in Sterile Insect Technique (SIT) (fig. S1) functioning similarly to sex-linked editors (*22*). Through modeling, we demonstrate that this technology may be suitable for release as a suppression tool, providing a scalable, self-limiting, and nondriving option for control of *A. gambiae* populations. The technology may also be adapted to other vector species to provide an alternative species-specific population suppression technology to control deadly disease vectors.

## RESULTS

### We engineered strains to target *fle*

**Fig. 1.**
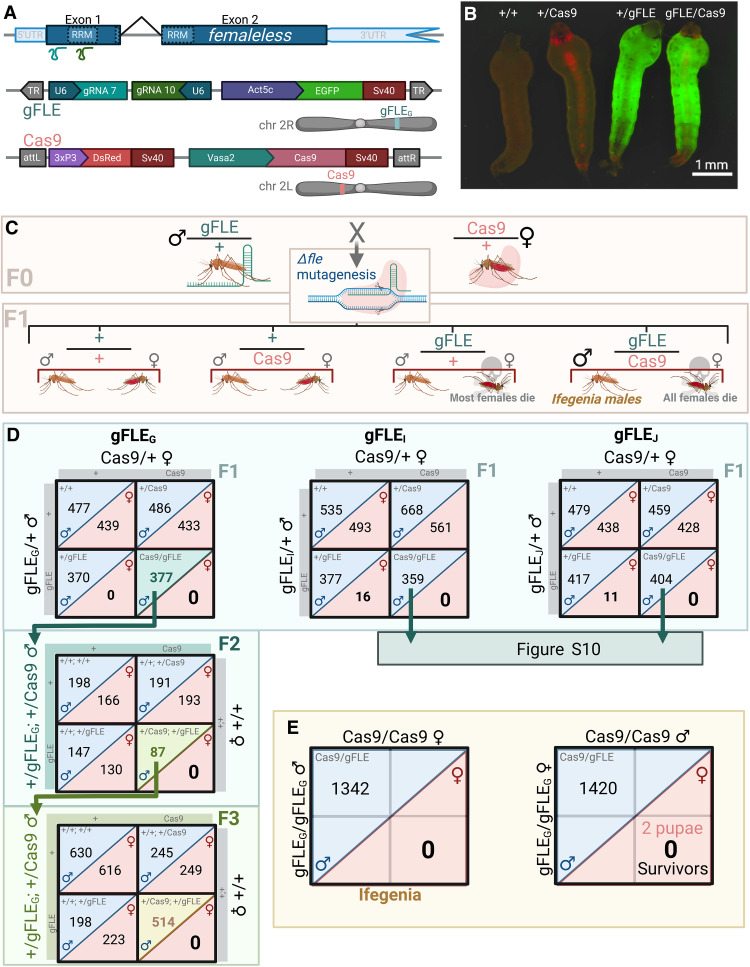
We developed a transgenic crossing system to target *fle* for mosaic knockout that results in robust female elimination. (**A**) The gFLE transgene expresses two gRNAs (gRNA7 and gRNA10, teal and emerald, respectively) targeting *fle* near the start codon and within the first RNA recognition motif (RRM), respectively (gene map, indigo). The PolIII U6 promoter facilitates gRNA expression (navy), selection is enabled by Act-EGFP-Sv40 (purple, green, and red), and the transgene was inserted by piggyBac transgenesis [terminal repeats (TR), gray arrows]. The Cas9 transgene expresses Cas9 in the adult germline and is maternally deposited into the embryo and was previously described by Werling *et al.* (*22*). (**B**) Individual genotypes are identified by fluorescence with Cas9 marked by 3xP3-DsRed and gFLE marked by Act5c-GFP. (**C**) Crossing F0 gFLE/+ males to Cas9/+ females yields F1 offspring in Mendelian 1:1:1:1 ratios, with the gFLE/Cas9 female cohort absent. (**D**) The absence of all F1 gFLE/Cas9 and some gFLE/+ female offspring suggests that *fle* mutagenesis results in female death. This effect is heritable into the F2 and F3 generations with gFLE_G_/Cas9 males mated to +/+ females able to kill daughters with the appropriate genotypes (F2 and F3 data from gFLE_I_/Cas9 and gFLE_J_/Cas9 males reported in fig. S10). (**E**) Crossing homozygous gFLE_G_/gFLE_G_ to Cas9/Cas9 results in complete elimination of adult genetic daughters from the progeny regardless of parental sex.

We hypothesized that embryonic biallelic CRISPR knockout of *fle* could cause female death. Therefore, to maintain line viability, we developed a bipartite system using separate gRNA and Cas9 lines to induce mosaic *fle* mutations in offspring (fig. S1). To ensure robust targeting, we designed and cloned a gRNA-expressing transgene encoding two gRNAs targeting the N-terminal region of *fle*; one targeting 25 base pairs (bp) downstream of the start codon, and a second targeting the first RNA recognition motif (Fig. 1A). We established three distinct transgenic families (termed gFLE_G_, gFLE_I_, and gFLE_J_) by piggyBac-mediated transgenesis and confirmed integration by *Act5C-GFP* selection. For Cas9 expression, we hypothesized that early embryonic and larval targeting of *fle* might yield strong female-killing effects. Therefore, we used Vasa2-Cas9 (hereon shortened to Cas9), provided by the Catteruccia Lab, for its robust maternal deposition of Cas9 protein into the embryo (Fig. 1A) (*23*).

### Disrupting *fle* kills females

To determine the phenotype of gFLE/Cas9 trans-heterozygotes, we performed preliminary genetic crosses between gFLE-positive males and Cas9-positive females of mixed hetero- and homozygosity and analyzed resulting offspring. Notably, all gFLE/Cas9 pupae scored, regardless of family, were phenotypic males (*n* = 638; table S1), indicating that Δ*fle* knockout resulted in robust loss of females. To confirm Δ*fle* mutagenesis , we sequenced gFLE/Cas9 adult males and identified Δ*fle* alleles at both gRNA target sites, although more under gRNA10 than gRNA7 (fig. S2).

Next, we sought to determine whether the loss-of-female Δ*fle* knockout phenotype was caused by female death or by androgenization that was sufficiently penetrant to make females phenotypically indistinguishable from males. To distinguish between these possibilities, we crossed gFLE/+ males to Cas9/+ females and quantified the offspring genotypes to observe any sex ratio deviating from the expected 50:50 within each Punnett genotype (Fig. 1, B and C). Notably, once again, no F1 gFLE/Cas9 adult genetic females were observed in any family during the course of all experiments and none in the F2 and F3 generations of the gFLE_G_/Cas9 family, despite 2512 gFLE/Cas9 males scored (tables S1, S2, S12, and S16 to S18). gFLE/Cas9 males (termed as Ifegenia males from hereon) were present at Mendelian ratios expected of linked transgenes, suggesting that gFLE/Cas9 females were killed rather than androgenized, as androgenization would have been observed as a doubling of gFLE/Cas9 individuals compared to other groups (Fig. 1D and table S2). Supporting this observation of female death rather than androgenization, polymerase chain reaction (PCR) confirmed the presence of a Y chromosome in all randomly selected Ifegenia individuals, suggesting that all gFLE/Cas9 individuals were genetically male (fig. S3). All three gFLE families showed a strong reduction in gFLE/+ females despite only receiving maternally deposited embryonic Cas9 protein: No gFLE_G_/+ females were identified, and a 23- and 38-fold reduction in females was observed among the gFLE_I_/+ (*n* = 377:16) and gFLE_J_/+ (*n* = 417:11) genotypes relative to male siblings, respectively (Fig. 1D and table S2). This suggests that disrupting *fle* alleles can kill gFLE/Cas9 inheriting females and that maternal deposition of Cas9 protein is sufficient for female killing in individuals inheriting gFLE alone.

Because gFLE_G_ yielded the strongest F1 hybrid female-killing phenotype among gFLE families, we proceeded with this line for further characterization. For this, we characterized transgene insertion sites and *fle* mutation profiles of gFLE_G_/Cas9 males by nanopore DNA sequencing, confirming a single-transgene insertion and expected Δ*fle* alleles (fig. S4). To determine relative *fle* expression, we performed RNA sequencing on three replicates of 18-hour-old embryos enriched for the gFLE_G_/Cas9 genotype compared to three replicates of each control genotype gFLE_G_, Cas9, and wild type (WT). We found a modest but highly significant reduction in *fle* (padj ranging from 4 × 10^–6^ to 2 × 10^–8^) (tables S3 to S10) with nonmutant genotypes clustering together as expected (fig. S5), with no observable change in *dsx*.

### *fle* is essential for female larval development

Next, to determine the life stage of female death, we quantified the genotypes of 1-day-old larvae from the cross outlined in Fig. 1C and did not observe a significant reduction in gFLE/Cas9 or gFLE/+ genotypes, indicating that the majority of gFLE/Cas9 females survive embryogenesis **(**fig. S6 and table S11). We then determined that most gFLE/Cas9 female death occurred during the larval stage by quantifying the survival of each sex-genotype from hatching to pupation (fig. S7 and table S12). Together, our results demonstrate that CRISPR targeting of *fle* has a strong larval female-killing phenotype.

### Ifegenia males are reproductively viable and competitive

To be candidates for release in population suppression campaigns, Ifegenia adult males must be long lived and reproductively viable. To quantify adult longevity, we monitored gFLE_G_/Cas9 and gFLE_J_/Cas9 males compared to +/+ siblings in survival assays. Male gFLE_G_/Cas9, but not gFLE_J_/Cas9, displayed slight reductions in longevity (fig. S8 and table S13). To verify reproductive viability, we crossed Ifegenia males to WT females and confirmed the presence of viable progeny (table S14). We then performed mating competition assays between equal numbers of Ifegenia and WT males, expecting 50% Ifegenia offspring if males were equally competitive. We observed nearly equal percent Ifegenia progeny as WT progeny (45.7%), indicating robust mating competitiveness (fig. S9 and table S15). Together, these results suggest that Ifegenia males should be sufficiently reproductively viable to achieve population suppression following iterative releases; however, large-scale cage and field trials should be undertaken in the future.

### Ifegenia induces genetic sexing

Given the profound female-killing properties of Ifegenia, we sought to determine its penetrance of genetic sexing for use in male-only vector control campaigns (fig. S1). We mated homozygous gFLE_G_/gFLE_G_ males to Cas9/Cas9 females, and vice versa, and verified the complete absence of females among all offspring (Fig. 1E and table S16). As expected, complete killing of genetic females was observed before pupal eclosure (*n* = 1342 gFLE_G_/Cas9 males from maternal Cas9 and *n* = 1420 males from paternal Cas9). However, we identified a single, likely sterile, phenotypic gFLE_G_/Cas9 female from the maternal Cas9 group as defined by the pupal genitalia parameters of our assay. This individual was PCR-amplified for the Y chromosome, revealing a rare male-feminization intersex genotype (table S16). Together, we have observed no evidence of viable F1 adult females in the gFLE_G_/Cas9 lines used in this work, revealing a powerful application of Ifegenia as a tool for genetic sexing of *A. gambiae* (fig. S1).

### Ifegenia males confer multigenerational daughter gynecide

We next sought to determine whether Ifegenia males can “load” nondriving CRISPR transgenes and Δ*fle* alleles into subsequent generations (F2 and F3) and cause multigenerational daughter-killing effects (Fig. 1D and figs. S2 and S10). We observed transmission of transgenes at ratios consistent with linkage of the gFLE and Cas9 transgenes on the second chromosome and complete female-killing of all F2 and F3 gFLE_G_/Cas9 females (Fig. 1D and tables S17 and S18). Moreover, out of the few F2 and F3 gFLE/Cas9 females observed from families gFLE_I_ and gFLE_J_, all either died as pupae or young adults, appeared androgenized, or were feminized XY genetic males. We also determined the approximate frequency of Δ*fle* alleles in the F2 generation by PCR and enzyme digest (*24*). We observed 85 to 95% Δ*fle* allele frequency among F2 individuals inheriting one or no transgenes, representing a conservative estimate on the frequency of germ cell mutagenesis in the parental F1 gFLE_G_/Cas9 germline. A number of individuals were sequenced for the mutant allele (fig. S2), providing evidence of heterozygous mutagenesis in *fle* (Δ*fle*), indicating that *fle* is likely haplosufficient to some degree, in contrast to earlier hypotheses that it was haplolethal (*20*). Among F2 progeny of the gFLE_G_/Cas9 genotype, 100% harbored at least one Δ*fle* allele; however, this is likely inflated due to active mosaic mutagenesis occurring in this genotype (fig. S11) (*13*, *25*). Together, these results indicate that Ifegenia males are reproductively viable and pass on both CRISPR transgenes and Δ*fle* alleles to subsequent generations, resulting in multigenerational daughter gynecide.

### Ifegenia induces confinable population suppression

**Fig. 2.**
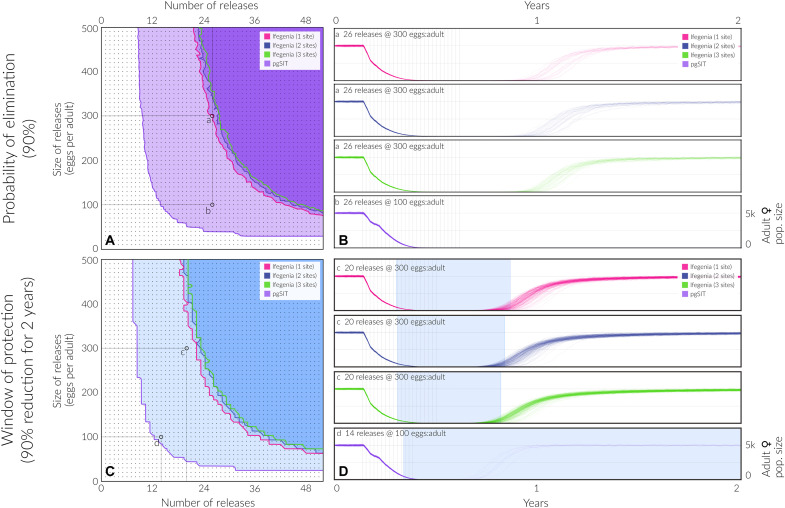
Model-predicted impact of releases of Ifegenia (one to three target sites) and pgSIT eggs on *A. gambiae* population density and elimination. Weekly releases were simulated in a randomly mixing population of 10,000 adult mosquitoes using the MGDrivE simulation framework (*27*) and parameters described in table S20. Weekly releases of up to 500 Ifegenia or pgSIT eggs per wild adult were simulated over 1 to 52 weeks. Ifegenia and pgSIT were simulated with cutting rates of 90% per allele, maternal deposition of Cas9 in 90% of embryos when expressed by the mother, and male mating competitiveness of 75% that of WT males. For Ifegenia, females homozygous for any Δ*fle* mutant allele were considered unviable, and, for pgSIT, males having the system were considered sterile. (**A**) Elimination probability is calculated as the percentage of 120 stochastic simulations that result in *A. gambiae* elimination, depicted for a range of release schemes: 1 to 52 consecutive weekly releases of 10 to 500 eggs per adult. Contours partition regions of parameter space that result in a ≥90% elimination probability for each system. (**B**) Illustrative time series are depicted for sample releases schemes. Scheme “a” depicts 26 releases of 300 eggs per wild adult, and scheme “b” depicts 26 releases of 100 eggs per wild adult. (**C**) Window of protection is calculated as the percentage of 120 stochastic simulations that result in the *A. gambiae* population being suppressed by ≥90% for ≥2 years, depicted for 1 to 52 consecutive weekly releases of 10 to 500 eggs per adult. Contours partition regions of parameter space that result in a window of protection of ≥2 years for each system. (**D**) Illustrative time series are depicted for sample releases schemes, with shaded regions representing the window of protection. Scheme “c” depicts 20 releases of 300 eggs per wild adult, and scheme “d” depicts 14 releases of 100 eggs per wild adult.

To determine whether iterative releases of fertile Ifegenia males could facilitate population suppression and elimination, we incorporated the above data on Ifegenia performance into a mathematical model (*26*) and simulated releases into a population of 10,000 *A. gambiae* adults (Fig. 2) with life history parameters described in table S20. Weekly releases of up to 500 Ifegenia eggs (female and male) per WT adult (female and male) were simulated over 1 to 52 weeks. The scale of these releases was chosen considering that adult release ratios of 10:1 are common for sterile male mosquito interventions (*27*) and female *A. gambiae* produce >30 eggs per day in temperate climates (*28*). We considered Ifegenia constructs with one to three gRNA target sites and, to be conservative, simulated target site cutting rates of 90% per allele, maternal deposition of Cas9 in 90% of embryos when expressed by the mother, and male mating competitiveness of 75% that of WT males. Results from these simulations suggest that substantial population suppression (≥90%) that endures for ≥2 years is observed for a wide range of achievable release schemes, e.g., >22 weekly releases of 300 or more Ifegenia eggs per wild adult, and elimination is expected to occur in ≥90% of simulations for >26 weekly releases of the same size. Ifegenia performance is robust to several system features—a sensitivity analysis revealed that the “window of protection” model outcome (the duration for which the population is suppressed by ≥90%) depends very little on the cutting rate, rate of maternal deposition of Cas, and mating competitiveness of males having the construct (fig. S12). Figure 2 reveals that the number of target sites also has little impact on modeled suppression outcomes; however, a supplementary analysis in which resistant allele generation is considered (text S1) reveals how increasing the number of target sites greatly reduces the rate at which resistant alleles propagate in the population (fig. S13). These findings suggest that Ifegenia can achieve robust temporary population suppression over a wide range of release parameter values, permitting rebound of native populations after ceasing releases. Opportunity for population rebound may be desired in locales where disease elimination has been achieved and where ecological concerns warrant return of the native mosquito, making Ifegenia a valuable vector control technique for the toolkit.

## DISCUSSION

In this work, we characterize mosaic CRISPR knockout of *fle* in *A. gambiae* and demonstrate its potential as a genetic sex sorting system and novel suppression technology. Our findings illustrate that using a bicomponent CRISPR system to target *fle* results in the complete killing of genetic females before eclosure through lethal mosaicism (*13*, *25*) and leaves F1 male siblings fit to mate and sire offspring (*29*, *30*). Termed Ifegenia, its strong female-killing phenotype makes it suitable for vector control campaigns that require male-only releases. Thus, we suggest two implementations for Ifegenia: (i) as a GSS to be combined with other vector control methods and (ii) as an independent population suppression vector control technology.

Historically, creating a scalable GSS in anophelines has been challenging, with many systems displaying low penetrance (*14*, *16*) or requiring manual or fluorescent-based sex sorting directly before release (*11*, *15*). Developing inducible genetic systems that automatically eliminate the undesired sex have been difficult due to limited understanding of the sex-determination pathway (*31*), and husbandry limitations have prohibited development of translocation lines (*4*). Overcoming these hurdles, Ifegenia’s design offers four key advantages that enable scalable production. (i) The two homozygous F0 stock strains have normal sex ratios and fertility, enabling mass rearing. (ii) A single F0 fertilized female yields conservatively 200 Ifegenia sons, facilitating large-scale production. (iii) The separation of gFLE and Cas9 stock lines prevents the creation of CRISPR-resistant alleles, circumventing roadblocks faced by some gene drives (*32*). (iv) The high phenotype penetrance enables direct release of F1 eggs/larvae/pupae into the wild, eliminating the need for injurious manual sorting and resources for rearing females. Where Ifegenia lacks scalability is the requirement for manual sorting to establish the F0 parental cross. A hurdle easily overcome by the addition of sex-specific fluorophores (*8*–*11*, *33*). Addition of sex-specific fluorophores in the F0 generation rather than the released F1 generation enables a much higher-throughput system. In this case, it would result in a sort:release ratio on the order of 1:50 (four adults yield one fertilized female that yields 200 lifetime sons) versus systems reliant on direct sorting of released males that yield a sort:release ratio closer to 2:1 (direct sorting of males from females). This would make Ifegenia’s throughput sufficient for most applications.

As an efficient and potentially scalable GSS, Ifegenia could help enable technologies like SIT in *A. gambiae* (*34*). SIT, which involves releasing sterilized males in mass for population suppression, has been successful in controlling several insect pests but has not yet been adapted to *A. gambiae* (*35*–*37*). After years of optimization, radiosterilization protocols that once reduced male fitness are now ready for larger trials, thus necessitating a male-only GSS in the species (*38*, *39*) and presenting a niche that could be fulfilled by Ifegenia.

In addition to serving as a GSS, Ifegenia could be used as a novel vector control technology. Our modeling suggests that iterative releases of Ifegenia males lead to long-term suppression in wild populations without drive following the introduction of CRISPR transgenes and female-killing alleles. We therefore propose that Ifegenia and other similar technologies (*22*) are an intermediate between control methods like pgSIT (*13*) and suppression gene drives (*17*, *40*). Unlike pgSIT, Ifegenia releases transgenes into the gene pool and induces multigenerational suppression, but, unlike gene drives, it is not self-propagating. This could make Ifegenia a desirable option in remote areas where repeated SIT releases are not feasible and gene drives have yet to gain acceptance. While resistant alleles could develop, this risk is reduced by targeting two different loci in *fle*. If resistant alleles persist, then a system with multiplexed targeting of additional female-essential genes could be developed, which, as modeling suggests, should achieve similar performance as targeting *fle* alone.

In this context, Ifegenia is comparable to female-specific flightless RIDL (fsRIDL), which similarly aims for male-only releases, induces female-killing, and has transgenes that persist for prolonged suppression. fsRIDL involves males passing down a repressible, toxic, and female-specific flight muscle transgene to their daughters, preventing them from flying. While fsRIDL has been successful in suppressing *Aedes* populations in trials (*41*) and is slated to be used in the United States (*42*), this design has not yet been reproduced in *A. gambiae.* Despite some differences in larval competition parameters and transgene number, its similarities with fsRIDL suggest that Ifegenia could achieve parallel outcomes in the field.

This study highlights the potential for *fle*-targeting technologies to enable new genetically modified vector control methods. High genetic conservation could permit trivial adaptation of Ifegenia and other *fle*-based tools to the related species *Anopheles arabiensis*, *Anopheles coluzzii*, and *Anopheles stephensi* (*20*) to curb their impact on malaria transmission. Ifegenia could facilitate further study of *fle*, yielding insights into its role in X-chromosome dosage compensation, and the biological function and of its homolog *Transformer2* (*43*), potentially enabling novel vector control systems in harmful agricultural pests such as *Ceratitis capitata* and *Lucilia cuprina* (*43*). Last, manipulating *fle* reveals opportunities for tet-based sex separation systems, homing-based gene drives (*44*–*46*), and Y drives (*47*) in addition to other tools, making possible a suite of new vector control technologies to target a range of pests. In all, Ifegenia not only provides important insights on the function of *fle* and demonstrates its value as a gene of interest for the vector control field at large but also provides a GSS and the first tool of its kind to combat malaria transmission in *A. gambiae.*

## MATERIALS AND METHODS

### Mosquito rearing and maintenance

*A. gambiae* was derived from the G3 strain. The mosquitoes were reared in 12-hour light/12-hour dark cycles at 27°C with 50 to 80% humidity in cages (Bugdorm, 24.5 cm by 24.5 cm by 24.5 cm) in an ACL-2 insectary. Adults were provided with 0.3 M aqueous sucrose ad libitum, and females were blood-fed on anesthetized mice for two consecutive days for ~15 min at a time. Males and females were allowed to mate for at least 2 days before a blood meal. Egg dishes were provided 2 days after a blood meal. Eggs were allowed to melanize for 2 days before being floated in trays. Larvae were reared and fed, as well as pupae screened and sexed, in accordance with established protocols (*48*).

### gRNA design, cloning, and transgenesis

The *fle* (AGAP013051) target gene reference sequence was extracted from VectorBase (*49*). To verify target sequence and detect any polymorphisms, gDNA was extracted (DNeasy Blood & Tissue Kits; Qiagen, catalog no./ID: 69504) from pools of 10 individuals, cloned into pJET (Thermo Fisher Scientific, catalog no./ID: K1231), and individual colonies were Sanger-sequenced for the *fle* locus. Putative candidate gRNAs in conserved regions were identified using http://crispor.tefor.net/. On-target in vitro cleavage activity was determined for the top 10 gRNA candidates by CRISPR QC (fig. S14). The gRNAs tested are as follows: gRNA1 (5′-TCATCCGCTTTCGACACTCG*CGG*-3′), gRNA2 (5′- CACGATGAGTATTGAGTCT*TGG*-3′), gRNA3 (5′-TGCTTCGTACAGCTGCCAGT*CGG*-3′), gRNA4 (5′-CTTCCACCGGCGGTAATCTT*TGG*-3′), gRNA5 (5′-TTATCACATTGTATGACGGT*CGG*-3′), gRNA6 (5′-GTGCTGGACGCATTCCTATT*GGG*-3′), gRNA7 (5′-CGACGGCTCGTTCATCGCTG*GGG-*3′), gRNA8 (5′-GTCGACGGCTCGTTCATCGC*TGG*-3′), gRNA 9 (5′-CTTGAACAGCTCTATCAGAT*CGG*-3′), and gRNA10 (5′-ATCGAGCGCGTCGCCTGGTA*CGG*-3′). Protospacer-adjacent motifs (PAMs) are shown in italics. Two gRNAs, gRNA7 and gRNA10, were selected as each targets exon 1 and overlap a semi-unique restriction enzyme site to facilitate downstream screening and identification of mutant alleles (*24*). We modified a piggyBac transgenesis backbone pbVTKactR (*50*) to contain each gRNA under expression of the *A. gambiae* U6 promoter (*23*) synthesized as gBlocks and replaced endogenous DsRed for enhanced green fluorescent protein (EGFP). The final plasmid sequence (plasmid 1154B, transgene gFLE) was confirmed by Sanger sequencing and is available on Addgene (no. 187238). Embryonic microinjections of gFLE into G3 WT embryos were carried out as described previously (*33*). We identified nine transgenic founders, which were individually outcrossed to WT to establish distinct families. Families gFLE_G_, gFLE_I_, and gFLE_J_ were selected for analysis.

### Fluorescent sorting, sexing, and imaging

*A. gambiae* was fluorescently sorted, sexed, and imaged using the Leica M165FC fluorescent stereomicroscope using a Leica DMC2900 camera. Fluorescence was visualized using the CFP/YFP/mCherry triple filter and was sexed by examination of pupal genital terminalia. In cases where sex was indeterminable by pupal phenotype, genotype was validated by Y-chromosome PCR (see below).

### Genetic cross setup

For all crosses, pupae were fluorescently sorted and sexed and allowed to emerge as adults in separate cages to ensure female virginity before crossing. Unless otherwise indicated, crosses were set up with 1- to 3-day old adults, allowed to mate ad libitum for 4 days, and then blood-fed. For preliminary test crosses, 50 gFLE-positive males and 50 Cas9-positive females (mixed heterozygotes and homozygotes) were crossed, and offspring pupal sex-genotypes were scored (table S1). For crosses requiring heterozygous parents, gFLE-positive or Cas9-positive individuals were first crossed to WT of the reciprocal sex and fluorescently sorted to generate guaranteed heterozygous F0s. These F0 +/gFLE males and +/Cas9 females were then intercrossed for analysis of the Mendelian inheritance patterns in the F1 offspring. For assays requiring homozygous × homozygous mating pairs, one of two assays was set up. In early experiments, small cages of gFLE-positive males (enriched for homozygotes by fluorescence intensity) were mated to pure Cas9/Cas9 females, allowed to mate en masse, and allowed to oviposit en masse. Only those broods that yielded 100% gFLE/Cas9 offspring were considered from gFLE/gFLE × Cas9/Cas9 and scored. In a second experiment, larger cross cages were set up essentially as described above; however, females were isolated into iso-female ovicups before oviposition. Individual broods were screened for pure hybrid transheterozygous (gFLE/Cas9) offspring, and only those with this genotype were scored.

### Embryo survival assays

To determine whether females were dying during embryogenesis, +/gFLE males and +/Cas9 females were crossed. From large egg lays, a random sampling of ~500 unhatched embryos were separated from the egg dish and allowed to hatch. All larvae were scored by genotype at 1 day after hatching and reported in fig. S6 and table S13.

### Larvae survival

To determine whether females were dying as larvae (after hatching and before pupation), we fluorescently sorted 1-day-old larval offspring of the +/gFLE × +/Cas9 cross. Forty larvae from each genotype were reared in separate trays, then scored as pupae by sex and genotype, and are reported in fig. S5 and table S14.

### *fle* knockout mutant analysis

DNA was individually extracted from L3 and L4 *A. gambiae* larvae using the DNeasy Blood & Tissue Kits (Qiagen, catalog no./ID: 69504). Genomic DNA (1 μl) was used as a template in a 20 μl of PCR reaction using Q5 HotStart DNA polymerase [New England Biolabs (NEB), catalog no./ID: M0493L] and primers 1154A.S5 and 1154A.S7 amplifying genomic *fle* sequences. The resulting product was run on a 1% agarose gel in tris-acetate-EDTA (TAE) buffer, gel-extracted with the Zymoclean Gel DNA Recovery Kit (Zymo Research, catalog no./ID: D4007), cloned into the pJET vector (Thermo Scientific, catalog no./ID: K1231), transformed into chemically competent *Escherichia coli* (Promega, JM109), and plated on LB-ampicillin plates. Sanger sequencing reads from individual colonies represented amplicons from individual mutant alleles, and primers PJET1-2F and/or PJET 1-2R were compared to *fle* sequences from our WT mosquitoes as a reference genome and a selection are summarized in fig. S2. All primer sequences can be found in table S21.

### Male mating competition assay

Competition cages were set up with 35 gFLE/Cas9 males × 35 WT males, or 70 WT males and then introduced into a cage with 35 WT virgin females. They were allowed to mate ad libitum for 5 days before bloodfeeding. Eggs were counted, and larvae were scored for the presence of a transgene. Because gFLE/Cas9 males sire some nontransgenic progeny as part of normal transgenic chromosome segregation, the ratio of transgenic:WT offspring was calculated in (fig. S9A) and used to calculate the WT offspring attributable to gFLE/Cas9 fathers in fig. S9B.

### Δ*fle* allele quantification

To quantify Δ*fle* mutant alleles, we performed PCR amplification followed by direct digest on *fle* amplicons in F2 individuals. Genomic DNA samples were prepared using the Qiagen DNeasy extraction kit (catalog no./ID: 69504) and amplified in 50 μl of PCR reactions using Taq DNA polymerase (NEB, catalog no./ID: M0273S) and primers 1154A.S8 and 1154A.S29 (1121 bp). PCR product was divided into three 15 μl of aliquots; one was undigested for reference, one was digested with BstNI (catalog no./ID: R0168S), and one was undigested with BseYI (NEB, catalog no. R0635S) according to the manufacturer’s protocols. PCR amplicons from WT alleles are expected to digest into 228- and 893-bp and into 403- and 718-bp fragments for BseY I (gRNA7) and BstN I (gRNA10), respectively. Failure to digest a significant quantity of PCR product indicates a likely polymorphism under the gRNA target site. Immediately after digestion, the 15-μl raw PCR product, 15-μl BstN I–digested product, and 15-μl BseY I–digested product were run side by side on gel [1% agarose gel in TAE buffer, 1-kb ladder (NEB, catalog no./ID: N3232L), most gels run at 115 V for 40 min]. Gel images shown in fig. S11.

### Y-chromosome amplification

Samples were extracted with the Qiagen DNeasy extraction kit (catalog no./ID: 69504). Genomic DNA (1 μl) was used as a template in a 20-μl PCR reaction using Q5 HotStart DNA polymerase (NEB, catalog no./ID: M0493L). Each individual was genotyped for the presence of the Y chromosome (*51*). Positive control PCRs used *A. gambiae*–specific 28*S* primers (1123A.S2/1123A.S3, 230-bp fragment) (*52*).

### Male survival curves

Ifegenia males and WT control males were scored as pupae and placed into separate cages. Cages were inspected each day for mosquito death. Dead adults were scored and removed daily, until no surviving adults remained.

### Mathematical modeling

To model the expected performance of Ifegenia and pgSIT at suppressing and eliminating local *A. gambiae* populations, we used the MGDrivE simulation framework (*26*). This framework models the egg, larval, pupal, and adult mosquito life stages with overlapping generations, larval mortality increasing with larval density, and a mating structure in which females retain the genetic material of the adult male with whom they mate for the duration of their adult life span. The inheritance patterns of the Ifegenia and pgSIT systems were modeled within the inheritance module of MGDrivE. For simplicity, the inheritance model assumes that mosquitoes having the Cas9 allele and a gRNA targeting the N-terminal region of *fle* or targeting male fertility in the case of pgSIT cleave recessive terminal regions of *fle* and/or of the male fertility allele according to a defined frequency. In the case of multiple target sites, cleavage of each target allele is treated as an independent event. For Ifegenia, females homozygous for any *fle* mutant allele have reduced viability, and, in the case of pgSIT, male mosquitoes homozygous for the fertility mutant allele have reduced fertility. A proportion of progeny of female mosquitoes having the gRNA and Cas9 alleles also have their N-terminal region of *fle* or, in the case of pgSIT, male fertility allele cleaved due to the maternal deposition of Cas9. For this analysis, no fitness effects are associated with having the gRNA or Cas9 alleles, except for male mating competitiveness, and individuals heterozygous for either allele are not affected. The development of resistance alleles, which could potentially inhibit the cleavage of gRNA target sites, is accounted for in a supplementary model (text S1).

We considered a single randomly mixing *A. gambiae* population of 10,000 mosquitoes and implemented the stochastic version of the MGDrivE framework to capture random effects at low population sizes and the potential for population elimination. Density-independent mortality rates for juvenile life stages were calculated for consistency with the population growth rate in the absence of density-dependent mortality, and density-dependent mortality was applied to the larval stage following Deredec *et al.* (*53*). Weekly releases of up to 500 transgenic eggs per wild adult mosquito (female and male) were simulated over a period of 1 to 52 weeks. The scale of egg releases was chosen following the precedent in (*25*) for equivalence to an adult release ratio on the order of 10:1, taking into account survival of released eggs through to the adult life stage in the presence of density-dependent larval mortality. A total of 120 repetitions were carried out for each parameter set, and mosquito genotype trajectories, along with the proportion of simulations that led to local population elimination, were recorded. Complete model and intervention parameters are listed in table S20.

### Determination of transgene integration sites

To determine the transgene insertion sites, we performed Oxford Nanopore genome DNA sequencing. We extracted genomic DNA using the Blood & Cell Culture DNA Midi Kit (Qiagen, catalog no./ID: 13343) from 16 adult Ifegenia transheterozygous males harboring both transgenes (Cas9/+; gFLE/+), following the manufacturer’s protocol. The sequencing library was prepared using the Oxford Nanopore SQK-LSK110 genomic library kit and sequenced on a single MinION flowcell (R9.4.1) for 72 hours. Base calling was performed with ONT Guppy base calling software version 6.1.2 using dna_r9.4.1_450bps_sup model generating 3.02 million reads above the quality threshold of *Q* ≧ 10 with N50 of 12,308 bp and total yield of 19.49 Gb. To identify transgene insertion sites, nanopore reads were aligned to plasmids carrying either gFLE (1154B, Addgene, as plasmid no. 187238) or Cas9 (*23*) constructs using minimap2 (*54*). Reads mapped to the plasmids were extracted and mapped to the *A. gambiae* genome (GCF_000005575.2_AgamP3). Exact insertion sites were determined by examining read alignments in Integrative Genomics Viewer. The gFLE_G_ transgene is integrated between positions 23,279,556 and 23,279,559 on chromosome 2R (NT_078266.2) and falls into the last intron of AGAP002582. Cas9 is inserted between positions 10,326,500 and 10,326,503 on 2L (NT_078265.2). The site is located in the intergenic region between AGAP005126 and AGAP005127 as expected by its integration in the X1 docking site (*33*). Using nanopore data, we also confirmed genomic deletions in the target gene, AGAP013051, as expected (fig. S4). The nanopore sequencing data has been deposited to the National Center for Biotechnology Information (NCBI) sequence read archive (PRJNA862928).

### Transcriptional profiling and expression analysis

To quantify target gene reduction and expression from transgenes and to assess global expression patterns, we performed Illumina RNA sequencing. A cross of 50 heavily enriched gFLE_G_/gFLE_G_ males to 50 homozygous Cas9/Cas9 females was performed to generate gFLE_G_/Cas9 offspring. Control WT, gFLE_G_-enriched, and Cas9 cages were also prepared. All cages were allowed to mate ad libitum for 5 days and were blood-fed. Seventy-two hours after the blood feeding, the egg dish is placed into cages for synchronous egg laying, and females allowed to oviposit for 2 hours. Eggs were collected 18 hours after the first egg lay was observed. We extracted total RNA using the miRNeasy Tissue/Cells Advanced Mini Kit (Qiagen, catalog no./ID: 217604) from 100-μl embryos, estimate volume, with each genotype (WT; +/Cas9; +/gFLE; gFLE/Cas9) in biological triplicate (12 samples total), following the manufacturer’s protocol. Genomic DNA was depleted using the gDNA eliminator column provided by the kit. RNA integrity was assessed using the RNA 6000 Pico Kit for Bioanalyzer (Agilent Technologies, catalog no./ID: 5067-1513), and mRNA was isolated from ~1 μg of total RNA using NEBNext Poly(A) mRNA Magnetic Isolation Module (NEB, catalog no./ID: E7490). RNA sequencing libraries were constructed using the NEBNext Ultra II RNA Library Prep Kit for Illumina (NEB, catalog no./ID: E7770) following the manufacturer’s protocols. Briefly, mRNA was fragmented to an average size of 200 nucleotides (nt) by incubating at 94°C for 15 min in the first-strand buffer. Complementary DNA was then synthesized using random primers and ProtoScript II Reverse Transcriptase followed by second-strand synthesis using the NEB Second Strand Synthesis Enzyme Mix. Resulting DNA fragments were end-repaired, dA-tailed, and ligated to NEBNext hairpin adaptors (NEB, catalog no./ID: E7335). Following ligation, adaptors were converted to the “Y” shape by treating with USER enzyme, and DNA fragments were size selected using Agencourt AMPure XP beads (Beckman Coulter, no. A63880) to generate fragment sizes between 250 and 350 bp. Adaptor-ligated DNA was PCR-amplified followed by AMPure XP bead cleanup. Libraries were quantified using the Qubit dsDNA HS Kit (Thermo Fisher Scientific, catalog no./ID: Q32854), and the size distribution was confirmed using the High Sensitivity DNA Kit for Bioanalyzer (Agilent Technologies, catalog no./ID: 5067-4626). Libraries were sequenced on an Illumina NextSeq2000 in paired-end mode with the read length of 50 nt and sequencing depth of 20 million reads per library. Base calls and FASTQ generation were performed with DRAGEN 3.8.4. The reads were mapped to the VectorBase-58_AgambiaePEST genome supplemented with gFLE and Cas9 transgene sequences using STAR. On average, ~97.4% of the reads were mapped (table S7). Gene expression was then quantified using featureCounts against VectorBase annotation release 58 GTF (VectorBase-58_AgambiaePEST.gtf). Transcript per million (TPM) values were calculated from counts produced by featureCounts and combined (table S8). Hierarchical clustering of the data shows that, for each genotype, all replicates cluster together, as expected (fig. S11). DESeq2 was then used to perform differential expression analyses between controls (WT; +/Cas9; +/gFLE) and gFLE/Cas9 (table S9 to S13). For each DESeq2 comparison, gene ontology enrichments were performed on significantly differentially expressed genes using R package topGO. Illumina RNA sequencing data have been deposited to the NCBI-SRA (PRJNA862928).

### Statistical analysis

Statistical analysis was performed in JMP8.0.2 by SAS Institute Inc. and Prism9 for macOS by GraphPad software LLC. At least three biological replicates were used to generate statistical means for comparisons. *P* values were calculated for a two-sided Student’s *t* test with equal or unequal variance. A two-sided *F* test was used to assess the variance equality. The departure significance for survival curves was assessed with the log-rank (Mantel-Cox) and Gehan-Breslow-Wilcoxon texts. Multiple comparisons were corrected by the Bonferroni method. All plots were constructed using Prism 9.1 for macOS by GraphPad software LLC.

## Supplementary Materials

### This PDF file includes:

Figs. S1 to S14 Text S1 Legends for tables S1 to S21 References
