# Supplementary material for: A confinable female-lethal population suppression system in the malaria vector, Anopheles gambiae
Source: Sci Adv. 2023 Jul 5;9(27):eade8903. doi: 10.1126/sciadv.ade8903 (PMC10321730; doi:10.1126/sciadv.ade8903)
Supplement: Supplementary file 1 — Figs. S1 to S14 Text S1 Legends for tables S1 to S21 References [file sciadv.ade8903_sm.pdf]

Science Advances
AAAS

Supplementary Materials for

A confinable female-lethal population suppression system in the malaria vector, Anopheles gambiae

Andrea L. Smidler *et al.*

Corresponding author: Andrea L. Smidler, James J. Pai, Reema A. Apte, Omar S. Akbari, oakbari@ucsd.edu

Sci. Adv. **9**, eade8903 (2023)
DOI: 10.1126/sciadv.ade8903

This PDF file includes:

Figs. S1 to S14
Text S1
Legends for tables S1 to S21
References

Other Supplementary Materials for this manuscript includes the following:

Tables S1 to S21

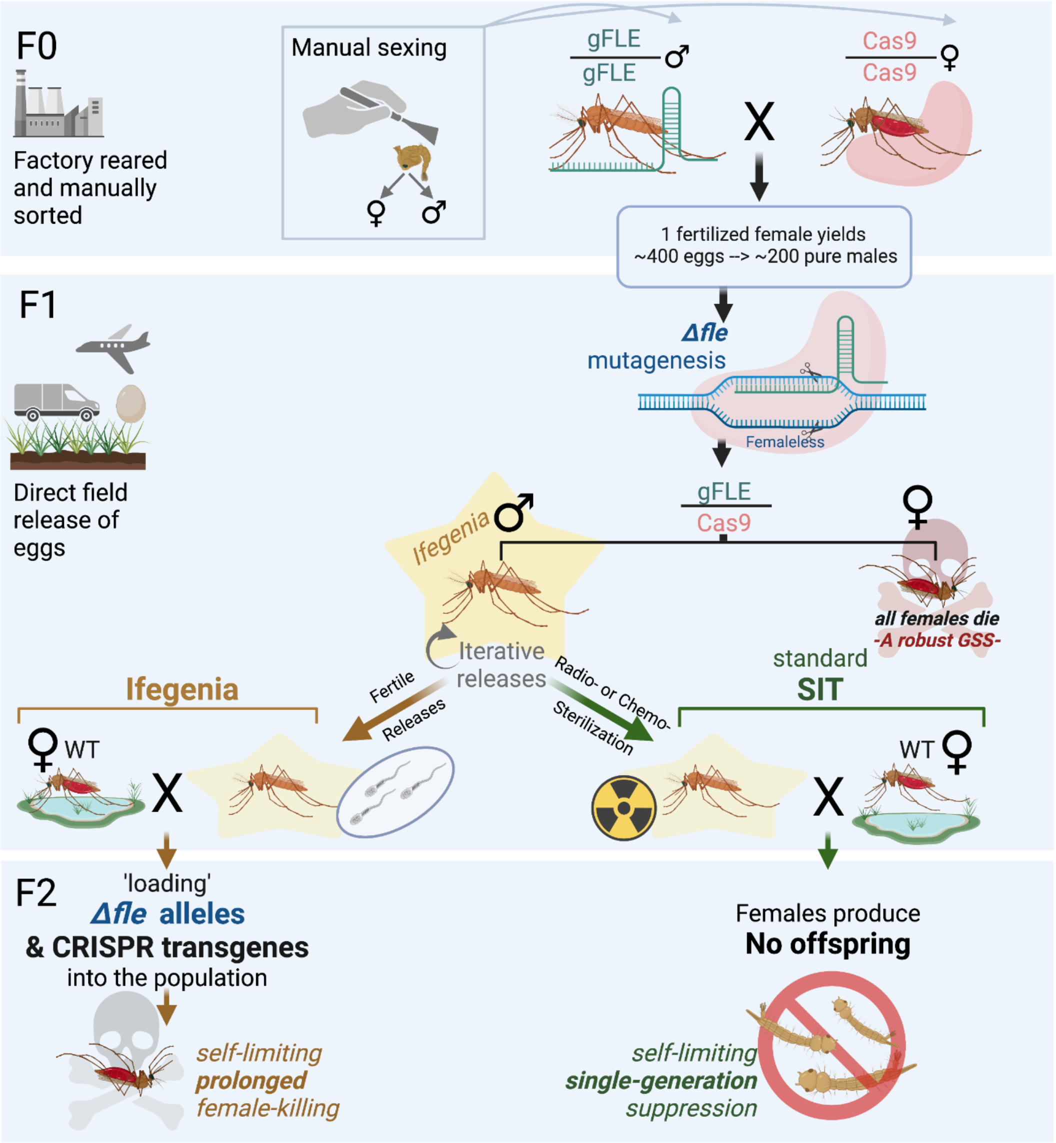

Figure S1. Using Ifegenia males for vector control. F0) Mass, factory-based rearing and sorting of males from a stock gFLE/gFLE line, and females from a Cas9/Cas9 line yields a F0 cross from which each fertilized female can produce ~200 Ifegenia sons during the course of her lifetime. **F1)** Produced F1 eggs which undergo significant *Δfle* mutagenesis are expected to be 100% Ifegenia males, producing a perfect Genetic Sexing Strain (GSS). Therefore these eggs can be directly released into the environment as part of an Ifegenia multi-generational suppression system (left), or sterilized for release as part of a more traditional SIT-based system (right). Once grown, the resulting F1 males will mate with wild type females to produce their respective population-suppression effects. **F2)** In a multi-generational suppression system, Ifegenia males remain fertile but 'load' *Δfle* alleles into the population to cause prolonged suppression by daughter killing - providing a non-driving technology. In a traditional SIT-based system involving male sterilization, no F2 offspring would result from released F1 Ifegenia males (right). Due to its profound daughter-killing phenotypes, we've termed it Ifegenia (**I**nherited **F**emale **E**limination by **G**enetically **E**ncoded **N**ucleases to **I**nterrupt **A**lleles), in honor of Iphigenia of Greek mythology who was sacrificed by her father, King Agamemnon, to win a great battle.

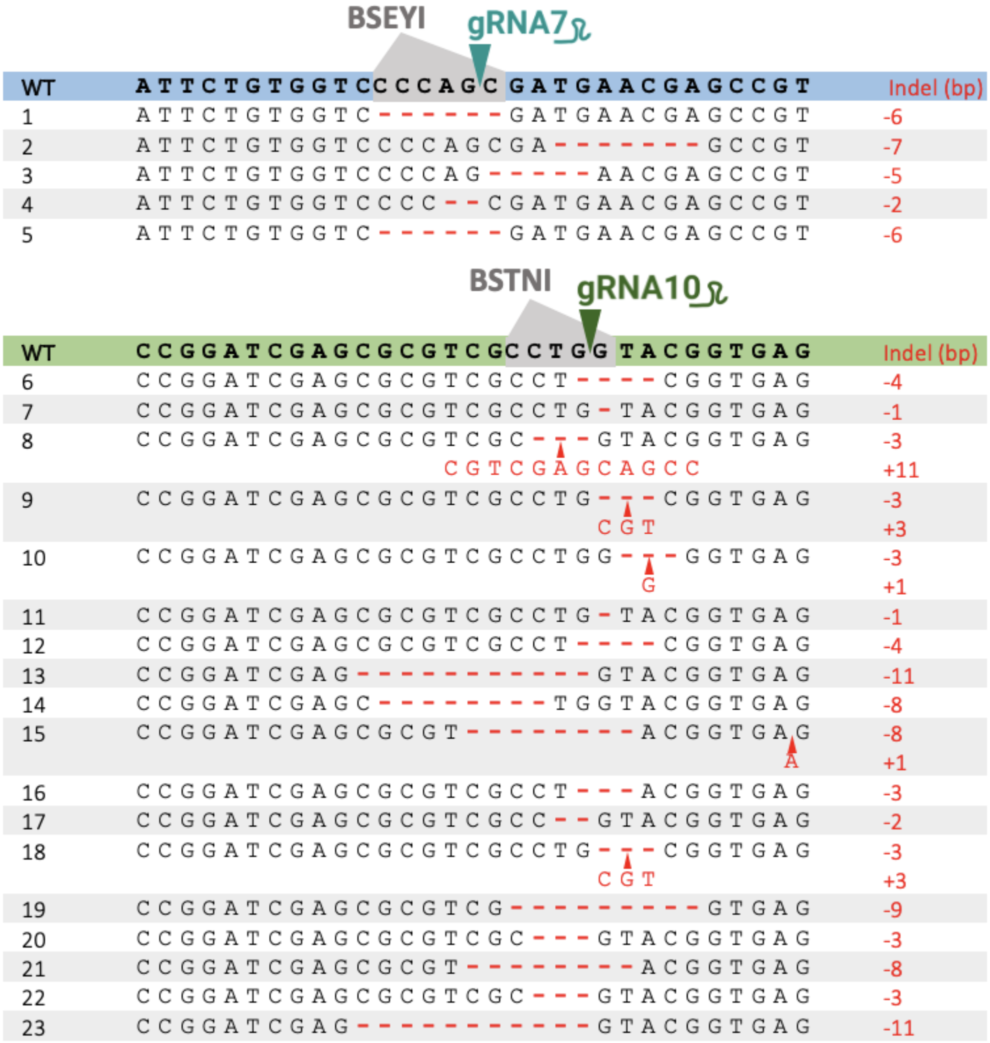

Figure S2: gFLE/Cas9 individuals have *Δfle* mutations under gRNA target sites.
gRNA7 (top): Reads 1-5 are from a $+_J/+$ larvae, $gFLE_G$/Cas9 adult male, $gFLE_J$/Cas9 larvae, a F2 $gFLE_I$/Cas9 phenotypic female that survived to adulthood, and a F3 $gFLE_J$/Cas9 intersex individual that died during eclosure respectively. **gRNA10 (bottom):** Reads 6-14 are from F2 larvae of mixed genotypes encompassing most genotypes and gFLE families. Read 15 was isolated from the only F1 $gFLE_G$/Cas9 phenotypic female identified (**Table S16**), however which later PCR amplified for the presence of a Y-chromosome suggesting feminization. Reads 16 and 17 were isolated from a F2 $gFLE_I$/Cas9 phenotypic female that survived to adulthood (**Table S17**), which also contained the gRNA7-derived mutation listed in Read 4. Reads 18 and 19 were identified from a F2 $gFLE_J$/Cas9 phenotypic female that died as a pupae. Reads 20 and 21 are from a F2 $gFLE_J$/Cas9 female that died as an adult. Reads 22 and 23 are from a F3 $gFLE_J$/Cas9 intersex individual that died during eclosure, which also contained gRNA7-derived Read 5.

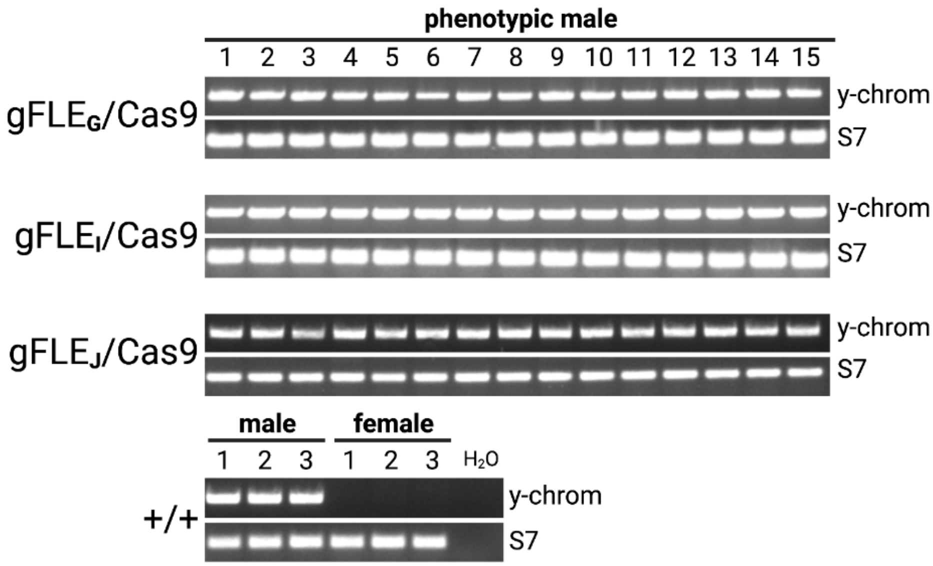

Figure S3. A random selection of gFLE/Cas9 transheterozygous adults which appeared phenotypically male were PCR amplified for the presence of the Y-chromosome.

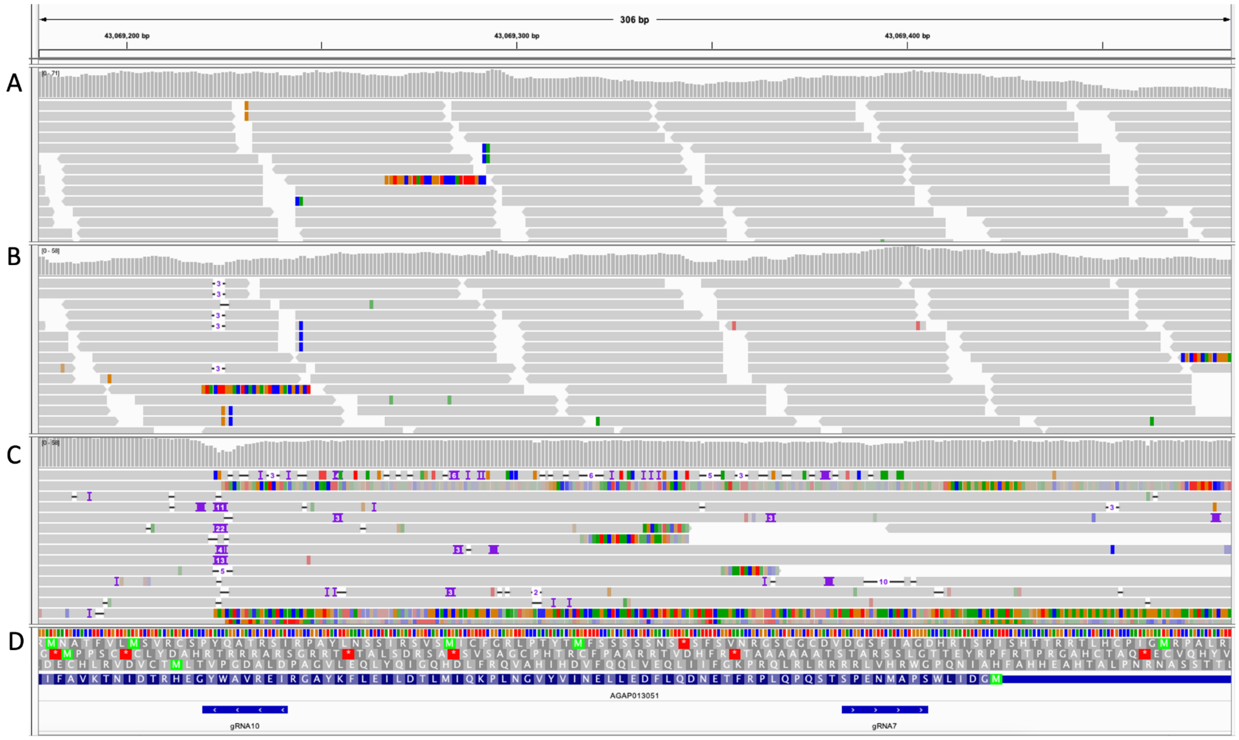

Figure S4. Genome browser snapshot of the *fle* gene (AGAP013051) zooming in on the gRNA target regions (2R:43,067,178-43,069,484). A) RNAseq reads of the WT control embryos. **B)** RNAseq reads of the gRNA/Cas9 transheterozygous embryos. **C)** Nanopore DNA sequencing of gRNA/Cas9 transheterozygous adult males. **D)** gRNA 10 and gRNA7 target sites indicated by blue bars.

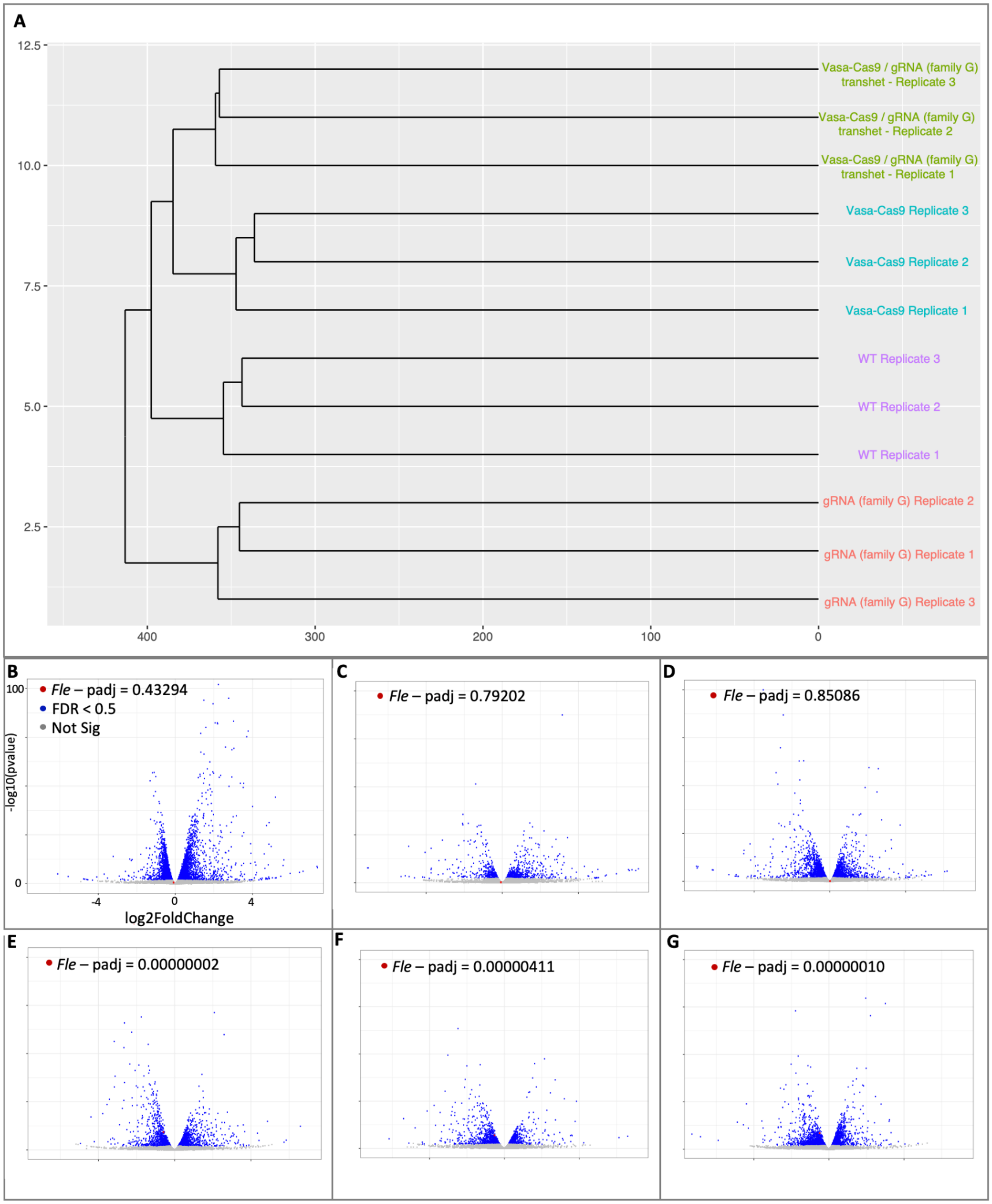

Figure S5. RNAseq samples clustering and their respective volcano plots. A) Hierarchical clustering of the 12 RNAseq samples. Volcano plots comparing: **B)** gRNA(Family G) replicates to the WT control replicates; **C)** Vasa-Cas9 replicates to the WT control replicates; **D)** Vasa-Cas9 replicates to the gRNA(Family G) replicates; **E)** Vasa-Cas9/gRNA(Family G) transhet replicates to WT control replicates; **F)** Vasa-Cas9/gRNA(Family G) transhet replicates to Vasa-Cas9 replicates; **G)** Vasa-Cas9/gRNA(Family G) transhet replicates to gRNA(Family G) replicates. The X and Y axis are the same for plots B-G.

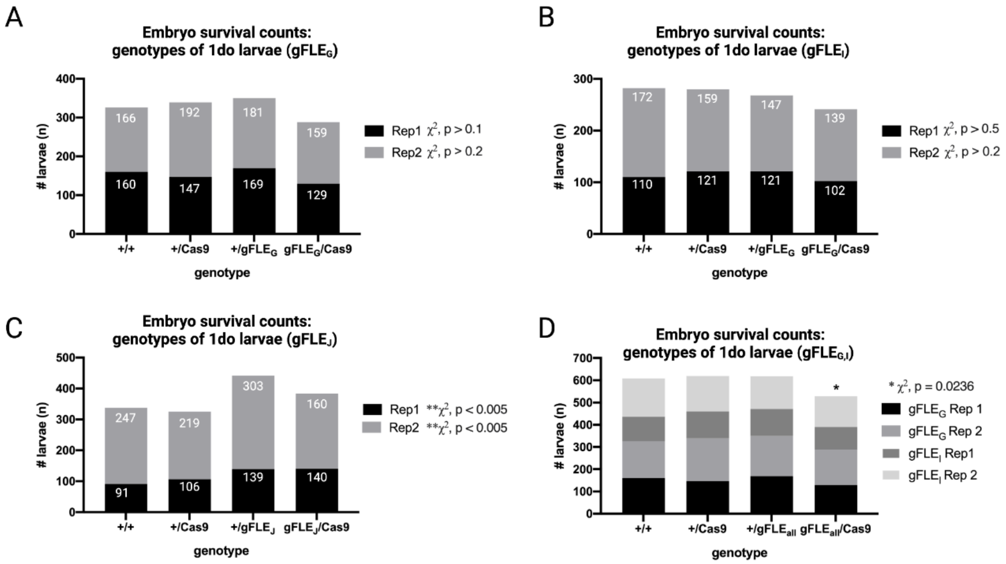

Figure S6. Mosaic mutant *Δfle* females survive embryogenesis. Reported as 1 day old (do) larvae counts by genotype of the offspring from a cross of gFLE/+ males to Cas9/+ females. Significant embryonic female death would be expected to be observed as an approximate halving of the gFLE/Cas9 group. **A)** Raw counts of 1do larvae by genotype, offspring from a +/gFLE$_G$ ♂ to +/Cas9♀cross. Two replicates shown stacked in black and grey. Both not significantly different from expected 1:1:1:1 Mendelian ratios, (χ^2, p > 0.1 and p > 0.2 respectively). **B)** 1do larvae counts from a +/gFLE$_I$♂ to +/Cas9♀ cross. Two replicates shown stacked in black and grey. Both are not significantly different from expected 1:1:1:1 Mendelian ratios, (χ^2, p > 0.5 and p > 0.2 respectively). **C)** 1do larvae counts from a +/gFLE$_J$♂ to +/Cas9♀ cross. Two replicates shown stacked in black and grey. Both significantly different from expected 1:1:1:1 Mendelian ratios (both χ^2, p < 0.005) consistent with multiple insertions of transgene gFLE$_J$. Family gFLE$_J$ was therefore omitted from the analysis in **D)**. **D)** Data pooled from **A)** and **B)**. Together these results demonstrate very slight levels of female embryo lethality in the gFLE/Cas9 group (χ^2 p = 0.0236)

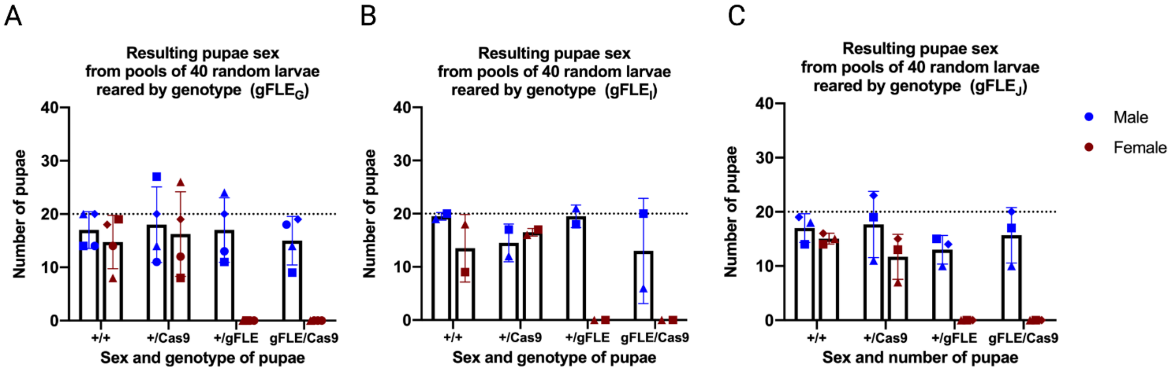

Figure S7. ***Femaleless*** **mosaic Δ*fle* mutants (gFLE/+ and gFLE/Cas9) die during larvaehood.** 40 random 1 day old (do) larvae were isolated into trays by genotype and reared separately. The number, sex, and genotype of individuals is reported upon pupation. Replicates 1-4 are denoted by triangle, square, diamond, and circle respectively. Mean and SD shown **A)** Δ*fle* females ($gFLE_G$/+ and $gFLE_G$/Cas9) were present at 1do but failed to pupate, all 4 replicates shown. **B)** Δ*fle* females ($gFLE_I$/+ and $gFLE_I$/Cas9) were present at 1do but failed to pupate. A third replicate was not performed on this line as the line was deemed sub-optimal for release and omitted from downstream analysis. **C)** Δ*fle* females ($gFLE_J$/+ and $gFLE_J$/Cas9) from family were present at 1do but failed to pupate, three replicates shown.

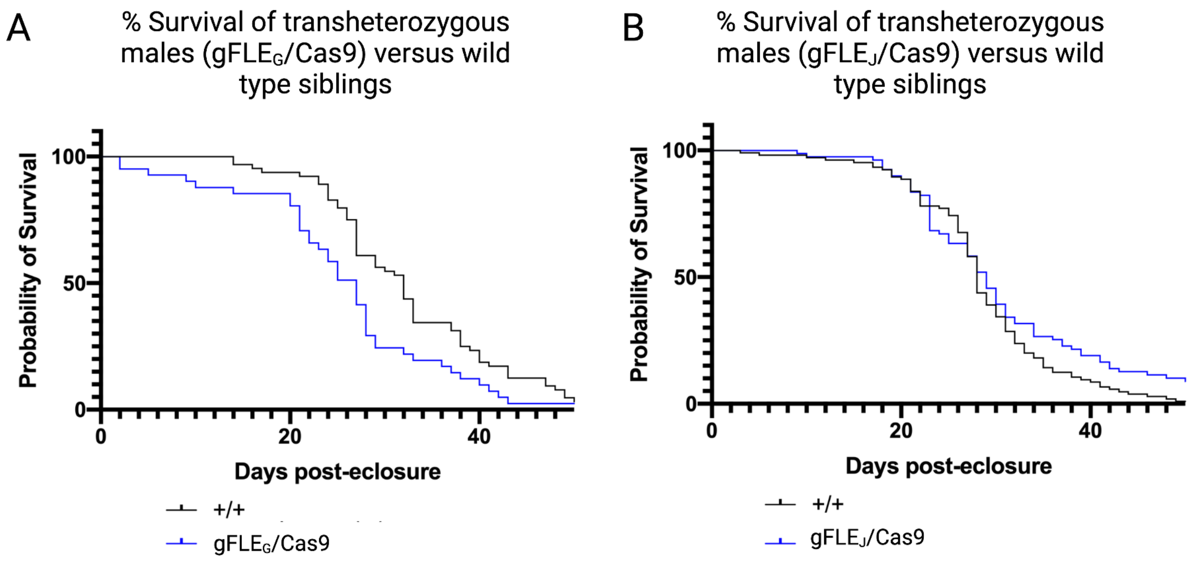

Figure S8. Male gFLE/Cas9 transheterozygotes die only slightly faster than wild type siblings. A) $gFLE_G$/Cas9 transheterozygous males (n = 40) die faster than wild type siblings (N = 74) (Log-rank p = 0.0074) **B)** $gFLE_J$/Cas9 transheterozygous males (n = 105) do not differ from wild type (n = 79) (Log-rank, p= 0.0558).

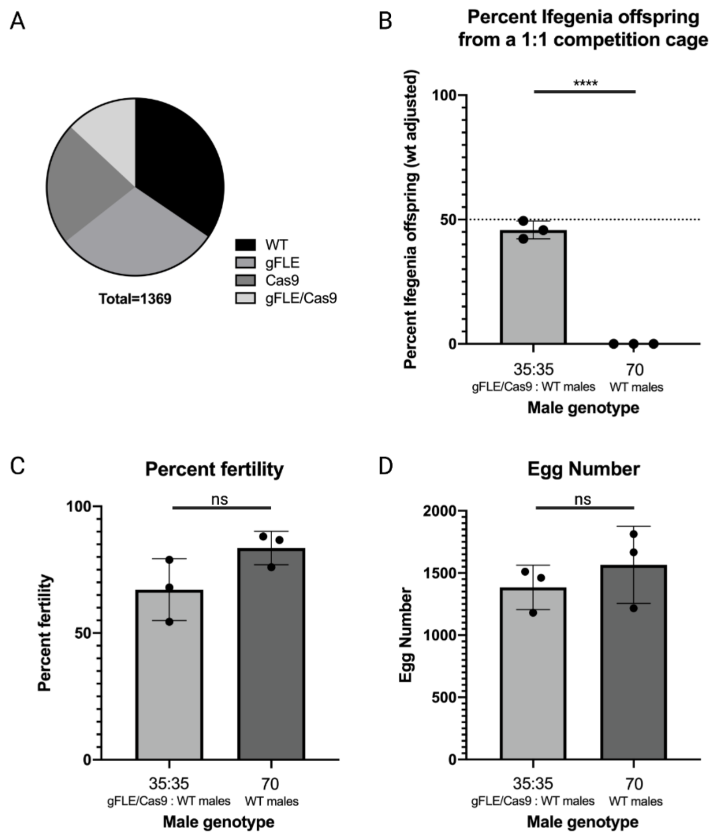

Figure S9. Ifegenia males have high mating competitiveness. **A)** Offspring genotype ratio from a cross of 35 Ifegenia males to 35 WT females. Genotype ratios were used to calculate the number of WT offspring in **(B)** that are attributed to Ifegenia males vs WT males. From this experiment it was determined that Ifegenia males sire 34.5% wild type offspring and 65.5% transgenics due to transgene linkage on the 2nd chromosome, instead of 25%/75% as would be expected if all transgenes were unlinked. **B)** Male mating competition assays of 35 Ifegenia males X 35 WT males X 35 WT females. All larvae were counted and genotyped. A fraction of WT larvae were attributed to Ifegenia fathers according to the ratios shown in **(A)** and reported with transgenic larvae as percent Ifegenia offspring (WT adjusted). Ifegenia positive larvae were calculated as ($n_{Ifegenia} = n_{transgenics} \times 1/0.655$), where 0.655 is the percent of transgenics from Ifegenia fathers as determined in **(A)**. Mean and SD shown. $p<0.0001$, unpaired two-tailed t-test **C)** Egg numbers produced by each replicate. Mean and SD shown, no significant difference unpaired t-test **D)** Hatching rate of each replicate. Mean and SD shown, no significant difference, unpaired t-test.

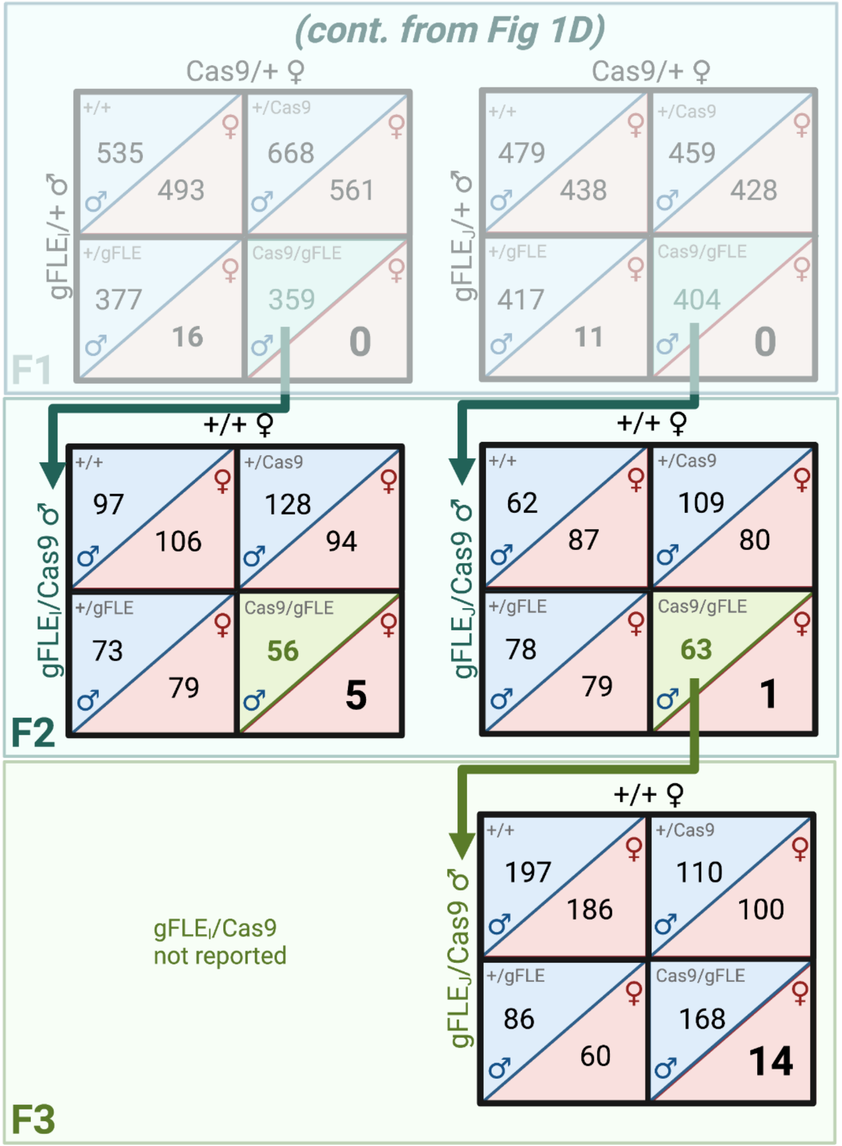

Figure S10. Female killing persists in F2 and F3 generations $gFLE_I$/Cas9 and $gFLE_J$/Cas9. In multigenerational experiments following from the crosses outlined in **Figure 1C**, F2 offspring from family $gFLE_I$ were followed through the F2 generation, and offspring from family $gFLE_J$ were followed through the F3 generation. Phenotypic gFLE/Cas9 females were identified in both families though at significantly reduced frequencies than should be expected by Mendelian segregation.

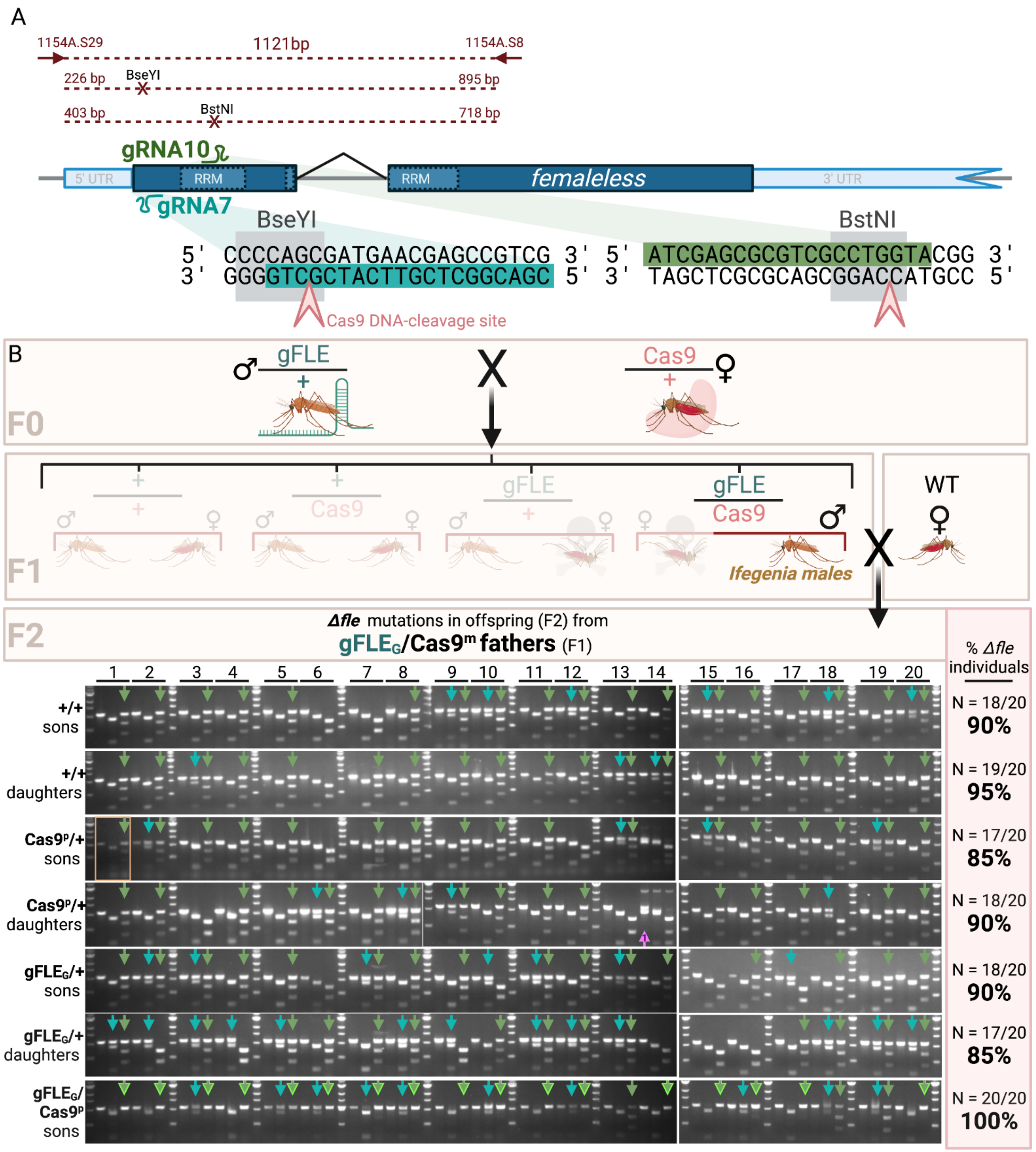

Figure S11. Approximate frequency of *Δfle* alleles in F2 offspring of gFLE$_G$/Cas9^m (F1) males. Hybrid F1 males inherited Cas9 maternally (Cas9^m) **A)** To quantify approximate knockout frequencies under the gRNA7 and gRNA 10 target sites, *fle* was PCR-amplified with primers 1154A.S29 and 1154A.S8 (red) and subsequently digested with either restriction enzyme BseYI (gRNA7) or BstNI (gRNA10). These enzymes have semi-unique recognition sites (gray boxes) in wild type overlapping the predicted CRISPR mutagenesis sites (salmon chevron). CRISPR mutagenesis will preclude enzyme digestion of the PCR amplicons corresponding to that allele for most mutations. In diploids, PCR of wild type sequences should fully digest, PCR of heterozygotes should partially digest, and PCR of homozygotes should fail to digest. **B)** 20 gFLE$_G$/Cas9 individuals for each genotype-sex are shown

numbered across the top in brackets. Three consecutive wells are loaded for each sample, left to right: undigested *fle* PCR product, BseYI-digested PCR product, BstNI-digested PCR product. Undigested BseYI and BstNI bands indicate probable CRISPR mutations under gRNA 7 (teal arrows) and gRNA 10 (olive arrows), respectively. Biallelic Δ*fle* mutants are noted (highlighted arrows), most of which are in the gFLE$_G$/Cas9 group due to active mosaic mutagenesis. Probable large insertions or deletions are denoted with purple arrows. Areas of the gel where exposure and brightness were adjusted separately are noted with orange boxes. The frequency of *Δfle* mutant individuals (those with at least one mutation) in that genotype-sex cohort are summarized at right in yellow.

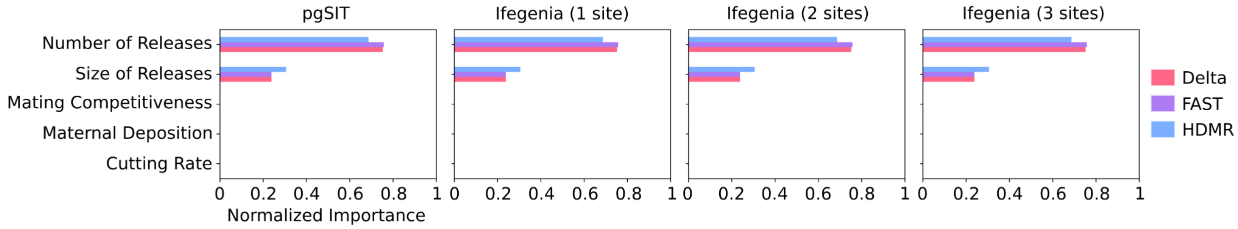

Figure S12. Sensitivity analysis of Ifegenia and pgSIT model outcomes to parameters describing the constructs, their fitness consequences and release schemes. Sensitivity analysis of the "window of protection" model outcome (i.e., the duration for which the *A. gambiae* population is suppressed by ≥90%) to parameter values describing the release scheme (number of releases, and number of eggs per release), fitness of transgenic mosquitoes (male mating competitiveness), and construct attributes (allelic cutting rate, and frequency of maternal deposition of Cas in embryos of mothers expressing Cas) for Ifegenia (1-3 sites) and pgSIT. The sensitivity analysis was conducted by simulating a randomly-mixing population of 10,000 adult mosquitoes using the MGDrivE simulation framework [27] with parameters described in **Table S20**. For parameters varied in the sensitivity analysis, number of releases varied from 1-48, number eggs released per adult varied from 10-500, male mating competitiveness varied from 0.5-1, allelic cutting rate varied from 0.75-1, and frequency of maternal deposition of Cas varied from 0.75-1. Parameter sensitivity was calculated based on three methods: i) Delta [54], ii) Fourier Amplitude Sensitivity Test (FAST) [55], and iii) High-Dimensional Model Representation (HDMR) [56]. According to all three sensitivity metrics, the window of protection outcome is most sensitive to parameter values describing the release scheme, and relatively insensitive to those describing the construct and mating competitiveness of transgenic mosquitoes.

Text S1. Modeling generation of alleles resistant to Ifegenia.

We calculate the efficiency of Ifegenia (N-target sites) at suppressing the propagation of generated resistant alleles. To this end, we first note that mosquito survival is a necessary condition for a newly-generated resistant allele to propagate to the next generation. Given the inheritance rules of Ifegenia, the resistant allele can only be passed to the next generation if the other $N - 1$ target sites are not cleaved on both chromosomes. Thus, we first calculate the probability, S, of a single target site not being cleaved on both chromosomes. This is the case when: i) no target site alleles are cleaved, ii) only one target site allele is cleaved, or iii) two alleles are cleaved and at least one of them develops resistance. Under these conditions, we calculate the probability of survival, S, as,

$$S = (1 - pC)^2 + 2pC(1 - pC) + pC^2(1 - (1 - pR^2).$$

Here, pC represents the probability that a target site is cleaved, given the presence of Cas and that the guide RNA that targets that site, and pR represents the probability that a resistant allele is generated at that target site, given it is cleaved. It follows from the above equation that, assuming cleavage of each target allele is an independent event, the probability that all N target sites are not cleaved on both chromosomes is given by S^N. This means that, beginning with a wild-type population, when a resistant allele emerges at one target site, the probability that the mosquito will survive and the resistant allele will be passed to the next generation is given by S^{N-1}.

We simulate Ifegenia with 2-4 target sites (**Figure S13**) and note that, for pC>90% and pR<10%, S^{N-1}<35%, 12% and 5%, respectively. In other words, the probability of propagating a resistant allele, assuming pC>90% and pR<10%, is reduced by more than 65%, 88% and 95% for Ifegenia with two, three and four target sites, respectively.

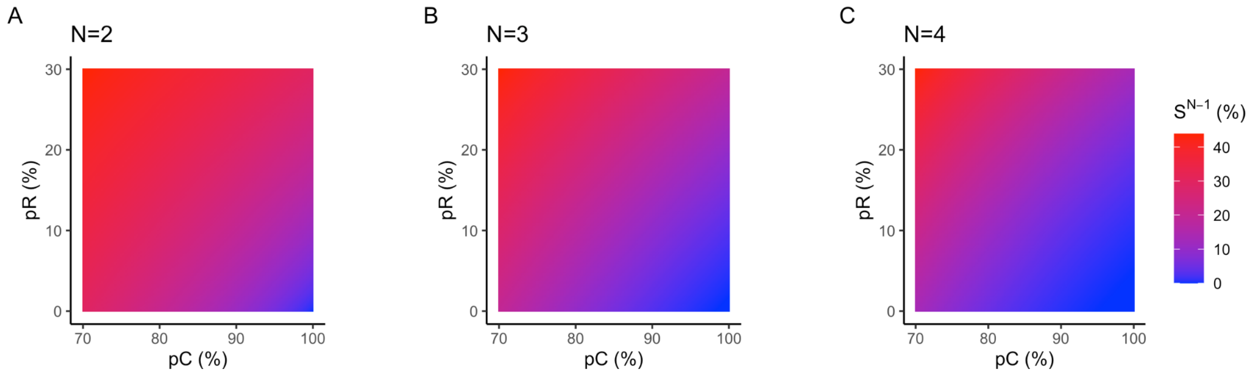

Figure S13. Mosquito survival probability given emergence of a resistant allele at one of the Ifegenia target sites. Survival probability is shown for Ifegenia having two **(A)**, three **(B)** and four **(C)** target sites, and is relative to the case for a single target site. The model used to derive mosquito survival probability is provided in **Text S1**. For cutting rate probability, pC>90%, and resistance generation probability, pR<10%, the mosquito survival probability, S^{N-1}, was found to be <35%, <12% and <5%, respectively. In other words, when the cutting rate is high (pC>90%) and emergence rate of resistance alleles is low (pR<10%), the probability of propagating a resistant allele from one generation to the next is reduced by more than 65%, 88% and 95% for Ifegenia having two, three and four target sites, respectively, as compared to the case of having one target site.

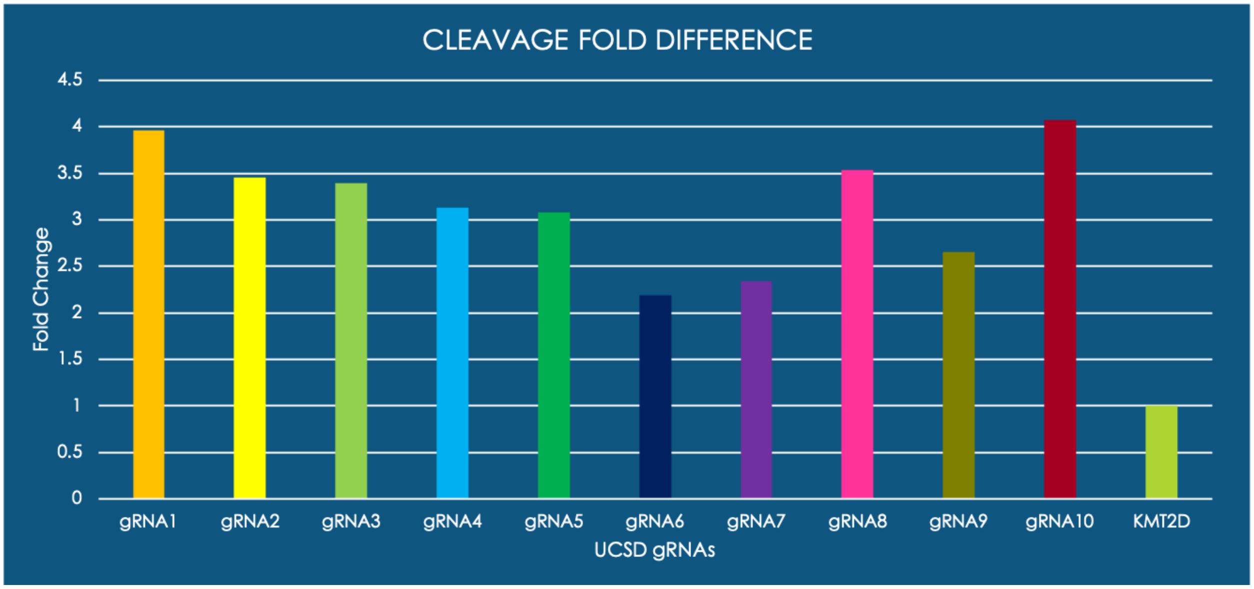

Figure S14. ***In vitro*** **gRNA cleavage assays performed by CRISPR QC.** Ten gRNAs predicted to be good candidates by http://crispor.tefor.net/ were assayed for cleavage efficacy using CRISPR QC's proprietary technology. Cleavage rates are reported as fold change in solution conductance normalized to KMT2D, a non-cutting gRNA with the template. gRNA7 and gRNA10 were selected for use in this work.

SUPPLEMENTARY TABLES

Table S1: Preliminary female-killing crosses. Raw sex-genotype pupae count for **F1** offspring from [+/gFLE & +/gFLE/gFLE] X [Cas9/Cas9 & +/Cas9] crosses.

Table S2: Raw sex-genotype pupae count for **F1** offspring from +/gFLE X +/Cas9 crosses. (Presented in **Fig 1D** Top row).

Table S3: RNAseq Mapping Stats for all 12 samples.

Table S4: Combined RNAseq TPM values for all 12 samples.

Table S5: RNAseq differential expression analysis comparing Vasa-*Cas9* vs. WT - control

Table S6: RNAseq differential expression analysis comparing gFLE(G) vs. WT

Table S7: RNAseq differential expression analysis comparing Vasa-*Cas9/*+ vs. gFLE(G)/+ transheterozygotes - control

Table S8: RNAseq differential expression analysis comparing Vasa-*Cas9* /gFLE(G) transheterozygotes vs. WT (+/+)

Table S9: RNAseq differential expression analysis comparing Vasa-*Cas9* /gFLE(G) transheterozygotes vs. Vasa-*Cas9*

Table S10: RNAseq differential expression analysis comparing Vasa-*Cas9* /gFLE(G) transheterozygotes vs. gFLE(G)

Table S11: Mendelian ratios of one day old larvae from +/gFLE X +/Cas9 - females survive embryogenesis.

Table S12: Survival of genotypes through larval stage - females die before pupation.

Table S13: gFLE/Cas9 adult male survival curves

Table S14: gFLE/Cas9 males bulk percent fertility.

Table S15: Male mating competition assays. Raw egg, larvae, genotype counts

Table S16: Raw sex-genotype pupae counts of **F1** offspring from homozygous $gFLE_G/gFLE_G$ males X Cas9/Cas9 females. (Presented in **Figure 1E**)

Table S17 : Raw sex-genotype pupae count for **F2** offspring from +/gFLE X +/Cas9 crosses. (Presented in; **Fig 1D** Bottom row left ($gFLE_G$), and **Fig S8** ($gFLE_I$, $gFLE_J$)

Table S18 : Raw sex-genotype pupae count for **F3** offspring from +/gFLE X +/Cas9 crosses. (Presented in; **Fig 1D** Bottom row right ($gFLE_G$), and **Fig S8** ($gFLE_I$, $gFLE_J$)

Table S19. Sex-genotype ratios of the F3 generation (offspring of F2 gFLE/Cas9 males x WT females) show that female-killing persists for multiple generations.

Table S20: Parameters used in *Anopheles gambiae* population suppression model.

Table S21: All Primers and gBlocks used in this study.

REFERENCES AND NOTES

1. W. H. Organization, Others, World malaria report 2020: 20 years of global progress and challenges (2020); https://apps.who.int/iris/bitstream/handle/10665/337660/9789240015791-eng.pdf.

2. H. Ranson, N. Lissenden, Insecticide resistance in african anopheles mosquitoes: A worsening situation that needs urgent action to maintain malaria control. *Trends Parasitol.* **32**, 187–196 (2016).

3. S. Sougoufara, E. C. Ottih, F. Tripet, The need for new vector control approaches targeting outdoor biting Anopheline malaria vector communities. *Parasit. Vectors* **13**, 295 (2020).

4. P. A. Papathanos, K. Bourtzis, F. Tripet, H. Bossin, J. F. Virginio, M. L. Capurro, M. C. Pedrosa, A. Guindo, L. Sylla, M. B. Coulibaly, F. A. Yao, P. S. Epopa, A. Diabate, A perspective on the need and current status of efficient sex separation methods for mosquito genetic control. *Parasit. Vectors* **11**, 654 (2018).

5. M. Zacarés, G. Salvador-Herranz, D. Almenar, C. Tur, R. Argilés, K. Bourtzis, H. Bossin, I. Pla, Exploring the potential of computer vision analysis of pupae size dimorphism for adaptive sex sorting systems of various vector mosquito species. *Parasit. Vectors* **11**, 656 (2018).

6. H. Yamada, M. J. B. Vreysen, K. Bourtzis, W. Tschirk, D. D. Chadee, J. R. L. Gilles, The *Anopheles arabiensis* genetic sexing strain ANO IPCL1 and its application potential for the sterile insect technique in integrated vector management programmes. *Acta Trop.* **142**, 138–144 (2015).

7. M. L. Taracena, C. M. Hunt, M. Q. Benedict, P. M. Pennington, E. M. Dotson, Downregulation of female doublesex expression by oral-mediated RNA interference reduces number and fitness of *Anopheles gambiae* adult females. *Parasit. Vectors* **12**, 170 (2019).

8. F. Bernardini, R. Galizi, M. Menichelli, P.-A. Papathanos, V. Dritsou, E. Marois, A. Crisanti, N. Windbichler, Site-specific genetic engineering of the *Anopheles gambiae* Y chromosome. *Proc. Natl. Acad. Sci. U.S.A.* **111**, 7600–7605 (2014).

9. F. Catteruccia, J. P. Benton, A. Crisanti, An *Anopheles* transgenic sexing strain for vector control. *Nat. Biotechnol.* **23**, 1414–1417 (2005).

10. A. L. Smidler, S. N. Scott, E. Mameli, W. R. Shaw, F. Catteruccia, A transgenic tool to assess *Anopheles* mating competitiveness in the field. *Parasit. Vectors* **11**, 651 (2018).

11. E. Marois, C. Scali, J. Soichot, C. Kappler, E. A. Levashina, F. Catteruccia, High-throughput sorting of mosquito larvae for laboratory studies and for future vector control interventions. *Malar. J.* **11**, 302 (2012).

12. G. Fu, R. S. Lees, D. Nimmo, D. Aw, L. Jin, P. Gray, T. U. Berendonk, H. White-Cooper, S. Scaife, H. Kim Phuc, O. Marinotti, N. Jasinskiene, A. A. James, L. Alphey, Female-specific flightless phenotype for mosquito control. *Proc. Natl. Acad. Sci. U.S.A.* **107**, 4550–4554 (2010).

13. M. Li, T. Yang, M. Bui, S. Gamez, T. Wise, N. P. Kandul, J. Liu, L. Alcantara, H. Lee, J. R. Edula, R. Raban, Y. Zhan, Y. Wang, N. DeBeaubien, J. Chen, H. M. Sánchez C., J. B. Bennett, I. Antoshechkin, C. Montell, J. M. Marshall, O. S. Akbari, Suppressing mosquito populations with precision guided sterile males. *Nat. Commun.* **12**, 5374 (2021).

14. R. Galizi, L. A. Doyle, M. Menichelli, F. Bernardini, A. Deredec, A. Burt, B. L. Stoddard, N. Windbichler, A. Crisanti, A synthetic sex ratio distortion system for the control of the human malaria mosquito. *Nat. Commun.* **5**, 3977 (2014).

15. F. A. Yao, A.-A. Millogo, P. S. Epopa, A. North, F. Noulin, K. Dao, M. Drabo, C. Guissou, S. Kekele, M. Namountougou, R. K. Ouedraogo, L. Pare, N. Barry, R. Sanou, H. Wandaogo, R. K. Dabire, A. McKemey, F. Tripet, A. Diabaté, Mark-release-recapture experiment in Burkina Faso demonstrates reduced fitness and dispersal of genetically-modified sterile malaria mosquitoes. *Nat. Commun.* **13**, 796 (2022).

16. E. Krzywinska, J. Krzywinski, Effects of stable ectopic expression of the primary sex determination gene Yob in the mosquito *Anopheles gambiae*. *Parasit. Vectors* **11**, 648 (2018).

17. K. Kyrou, A. M. Hammond, R. Galizi, N. Kranjc, A. Burt, A. K. Beaghton, T. Nolan, A. Crisanti, A CRISPR–Cas9 gene drive targeting doublesex causes complete population suppression in caged *Anopheles gambiae* mosquitoes. *Nat. Biotechnol.* **36**, 1062–1066 (2018).

18. K. C. Long, L. Alphey, G. J. Annas, C. S. Bloss, K. J. Campbell, J. Champer, C.-H. Chen, A. Choudhary, G. M. Church, J. P. Collins, K. L. Cooper, J. A. Delborne, O. R. Edwards, C. I. Emerson, K. Esvelt, S. W. Evans, R. M. Friedman, V. M. Gantz, F. Gould, S. Hartley, E. Heitman, J. Hemingway, H. Kanuka, J. Kuzma, J. V. Lavery, Y. Lee, M. Lorenzen, J. E. Lunshof, J. M. Marshall, P. W. Messer, C. Montell, K. A. Oye, M. J. Palmer, P. A. Papathanos, P. N. Paradkar, A. J. Piaggio, J. L. Rasgon, G. Rašić, L. Rudenko, J. R. Saah, M. J. Scott, J. T. Sutton, A. E. Vorsino, O. S. Akbari, Core commitments for field trials of gene drive organisms. *Science* **370**, 1417–1419 (2020).

19. A. K. Beaghton, A. Hammond, T. Nolan, A. Crisanti, A. Burt, Gene drive for population genetic control: Non-functional resistance and parental effects. *Proc. Biol. Sci.* **286**, 20191586 (2019).

20. E. Krzywinska, L. Ferretti, J. Li, J.-C. Li, C.-H. Chen, J. Krzywinski, *femaleless* controls sex determination and dosage compensation pathways in females of *Anopheles* mosquitoes. *Curr. Biol.* **31**, 1084–1091.e4 (2021).

21. O. Akbari, M. Li, N. Kandul, R. Sun, T. Yang, E. D. Benetta, D. Brogan, I. Antoshechkin, O. Akbari, M. Li, N. Kandul, R. Sun, T. Yang, E. D. Benetta, D. Brogan, I. Antoshechkin, Héctor Sánchez C., Y. P. Zhan, N. De Beaubien, Y. M. Loh, M. Su, C. Montell, J. Marshall, Targeting sex determination to suppress mosquito populations. *Res. Sq*, rs-2834069 (2023).

22. A. Burt, A. Deredec, Self-limiting population genetic control with sex-linked genome editors. *Proc. Biol. Sci.* **285**, 20180776 (2018).

23. K. Werling, W. R. Shaw, M. A. Itoe, K. A. Westervelt, P. Marcenac, D. G. Paton, D. Peng, N. Singh, A. L. Smidler, A. South, A. A. Deik, L. Mancio-Silva, A. R. Demas, S. March, E. Calvo, S. N. Bhatia, C. B. Clish, F. Catteruccia, Steroid hormone function controls non-competitive plasmodium development in anopheles. *Cell* **177**, 315–325.e14 (2019).

24. A. L. Smidler, O. Terenzi, J. Soichot, E. A. Levashina, E. Marois, Targeted mutagenesis in the malaria mosquito using TALE nucleases. *PLOS ONE* **8**, e74511 (2013).

25. N. P. Kandul, J. Liu, H. M. Sanchez C., S. L. Wu, J. M. Marshall, O. S. Akbari, Transforming insect population control with precision guided sterile males with demonstration in flies. *Nat. Commun.* **10**, 84 (2019).

26. Héctor M. Sánchez C., S. L. Wu, J. B. Bennett, J. M. Marshall, MGDriv E: A modular simulation framework for the spread of gene drives through spatially explicit mosquito populations. *Methods Ecol. Evol.***11**, 229–239 (2020).

27. D. O. Carvalho, A. R. McKemey, L. Garziera, R. Lacroix, C. A. Donnelly, L. Alphey, A. Malavasi, M. L. Capurro, Suppression of a field population of aedes aegypti in Brazil by sustained release of transgenic male mosquitoes. *PLoS Negl. Trop. Dis.* **9**, e0003864 (2015).

28. J.-M. O. Depinay, C. M. Mbogo, G. Killeen, B. Knols, J. Beier, J. Carlson, J. Dushoff, P. Billingsley, H. Mwambi, J. Githure, A. M. Toure, F. E. McKenzie, A simulation model of African *Anopheles* ecology and population dynamics for the analysis of malaria transmission. *Malar. J.* **3**, 29 (2004).

29. G. M. Chambers, M. J. Klowden, Age of Anopheles gambiae Giles male mosquitoes at time of mating influences female oviposition. *J. Vector Ecol.* **26**, 196–201 (2001).

30. S. P. Sawadogo, A. Diabaté, H. K. Toé, A. Sanon, T. Lefevre, T. Baldet, J. Gilles, F. Simard, G. Gibson, S. Sinkins, R. K. Dabiré, Effects of age and size on *Anopheles gambiae* s.s. Male mosquito mating success. *J. Med. Surg. Pathol.* 285–293 (2013).

31. E. Krzywinska, N. J. Dennison, G. J. Lycett, J. Krzywinski, A maleness gene in the malaria mosquito *Anopheles gambiae*. *Science***353**, 67–69 (2016).

32. R. Raban, W. A. C. Gendron, O. S. Akbari, A perspective on the expansion of the genetic technologies to support the control of neglected vector-borne diseases and conservation. *Front. Trop. Dis.* **3**, 999273 (2022).

33. G. Volohonsky, O. Terenzi, J. Soichot, D. A. Naujoks, T. Nolan, N. Windbichler, D. Kapps, A. L. Smidler, A. Vittu, G. Costa, S. Steinert, E. A. Levashina, S. A. Blandin, E. Marois, Tools for *Anopheles gambiae* Transgenesis. *G3 (Bethesda)* **5**, 1151–1163 (2015).

34. E. F. Knipling, Sterile-male method of population control. *Science* **130**, 902–904 (1959).

35. C. Concha, A. Palavesam, F. D. Guerrero, A. Sagel, F. Li, J. A. Osborne, Y. Hernandez, T. Pardo, G. Quintero, M. Vasquez, G. P. Keller, P. L. Phillips, J. B. Welch, W. O. McMillan, S. R. Skoda, M. J. Scott, A transgenic male-only strain of the New World screwworm for an improved control program using the sterile insect technique. *BMC Biol.* **14**, 72 (2016).

36. T. Ant, M. Koukidou, P. Rempoulakis, H.-F. Gong, A. Economopoulos, J. Vontas, L. Alphey, Control of the olive fruit fly using genetics-enhanced sterile insect technique. *BMC Biol.* **10**, 51 (2012).

37. J. Hendrichs, G. Franz, P. Rendon, Increased effectiveness and applicability of the sterile insect technique through male-only releases for control of Mediterranean fruit flies during fruiting seasons. *J. Appl. Entomol.* **119**, 371–377 (1995).

38. G. Munhenga, B. D. Brooke, J. R. L. Gilles, K. Slabbert, A. Kemp, L. C. Dandalo, O. R. Wood, L. N. Lobb, D. Govender, M. Renke, L. L. Koekemoer, Mating competitiveness of sterile genetic sexing strain males (GAMA) under laboratory and semi-field conditions: Steps towards the use of the Sterile Insect Technique to control the major malaria vector Anopheles arabiensis in South Africa. *Parasit. Vectors* **9**, 122 (2016).

39. K. Bourtzis, Z. J. Tu, Joint FAO/IAEA Coordinated Research Project on "Exploring genetic, molecular, mechanical and behavioural methods of sex separation in mosquitoes" – An introduction. *Parasit. Vectors* **11**, 653 (2018).

40. A. Hammond, R. Galizi, K. Kyrou, A. Simoni, C. Siniscalchi, D. Katsanos, M. Gribble, D. Baker, E. Marois, S. Russell, A. Burt, N. Windbichler, A. Crisanti, T. Nolan, A CRISPR-Cas9 gene drive system targeting female reproduction in the malaria mosquito vector *Anopheles gambiae*. *Nat. Biotechnol.* **34**, 78–83 (2016).

41. S. Halliday, Oxitec successfully completes first field deployment of 2nd Generation friendly Aedes aegypti technology. *Oxitec* (2019); www.oxitec.com/en/news/oxitec-successfully-completes-first-field-deployment-of-2nd-generation-friendly-aedes-aegypti-technology.

42. J. Romeis, J. Collatz, D. C. M. Glandorf, M. B. Bonsall, The value of existing regulatory frameworks for the environmental risk assessment of agricultural pest control using gene drives. *Environ. Sci. Policy* **108**, 19–36 (2020).

43. M. Scott, Sex determination and dosage compensation: Femaleless is the link in anopheles mosquitoes. *Curr. Biol.* **31**, R260–R263 (2021).

44. K. M. Esvelt, A. L. Smidler, F. Catteruccia, G. M. Church, Concerning RNA-guided gene drives for the alteration of wild populations. *eLife* **3**, e03401 (2014).

45. J. Champer, A. Buchman, O. S. Akbari, Cheating evolution: Engineering gene drives to manipulate the fate of wild populations. *Nat. Rev. Genet.* **17**, 146–159 (2016).

46. N. P. Kandul, J. Liu, J. B. Bennett, J. M. Marshall, O. S. Akbari, A confinable home-and-rescue gene drive for population modification. *eLife* **10**, e65939 (2021).

47. A. Simoni, A. M. Hammond, A. K. Beaghton, R. Galizi, C. Taxiarchi, K. Kyrou, D. Meacci, M. Gribble, G. Morselli, A. Burt, T. Nolan, A. Crisanti, A male-biased sex-distorter gene drive for the human malaria vector Anopheles gambiae. *Nat. Biotechnol.* **38**, 1054–1060 (2020).

48. M. Q. Benedict, The MR4 Methods in Anopheles research laboratory manual. Atlanta: CDC; 2007 (2018).

49. G. I. Giraldo-Calderón, S. J. Emrich, R. M. MacCallum, G. Maslen, E. Dialynas, P. Topalis, N. Ho, S. Gesing; VectorBase Consortium, G. Madey, F. H. Collins, D. Lawson, An updated bioinformatics resource for invertebrate vectors and other organisms related with human diseases. *Nucleic Acids Mol. Biol.* **43**, D707–D713 (2015).

50. A. Smidler, "CRISPR-based innovative genetic tools for control of *Anopheles gambiae* mosquitoes." thesis, Harvard University (2019); https://dash.harvard.edu/handle/1/42029729.

51. J. Krzywinski, D. R. Nusskern, M. K. Kern, N. J. Besansky, Isolation and characterization of Y chromosome sequences from the African malaria mosquito Anopheles gambiae. *Genetics* **166**, 1291–1302 (2004).

52. R. J. Kent, A. J. West, D. E. Norris, Molecular differentiation of colonized human malaria vectors by 28S ribosomal DNA polymorphisms. *Am. J. Trop. Med. Hyg.* **71**, 514–517 (2004).

53. A. Deredec, H. C. J. Godfray, A. Burt, Requirements for effective malaria control with homing endonuclease genes. *Proc. Natl. Acad. Sci. U.S.A.***108**, E874–E880 (2011).

54. H. Li, Minimap2: Pairwise alignment for nucleotide sequences. *Bioinformatics* **34**, 3094–3100 (2018).

55. E. Borgonovo, A new uncertainty importance measure. *Reliab. Eng. Syst. Saf.* **92**, 771–784 (2007).

56. R. I. Cukier, C. M. Fortuin, K. E. Shuler, A. G. Petschek, J. H. Schaibly, Study of the sensitivity of coupled reaction systems to uncertainties in rate coefficients. I Theory. *J. Chem. Phys.* **59**, 3873–3878 (1973).

57. A. Saltelli, S. Tarantola, K. P.-S. Chan, A quantitative model-independent method for global sensitivity analysis of model output. *Dent. Tech.* **41**, 39–56 (1999).

58. World Health Organization (WHO), *Garki project: Research on the epidemiology and control of malaria in the Sudan Savanna of west Africa* (World Health Organization, Genève, Switzerland, 1980); https://apps.who.int/iris/handle/10665/40316.

59. C. Taylor, Y. T. Touré, J. Carnahan, D. E. Norris, G. Dolo, S. F. Traoré, F. E. Edillo, G. C. Lanzaro, Gene flow among populations of the malaria vector, *Anopheles gambiae*, in Mali, West Africa. *Genetics* **157**, 743–750 (2001).
